# SLMO2 is a potential prognostic and immunological biomarker in human pan-cancer

**DOI:** 10.1038/s41598-024-51720-5

**Published:** 2024-01-11

**Authors:** Xiong Liu, Renming Yuan, Jie Peng, Ailei Xu, Xiaoxia Nie, Ruiti Tang, Guangqiang Li

**Affiliations:** 1 Clinical Laboratory, Hunan Aerospace Hospital, 189 Fenglin 3rd Road, Yuelu District, Changsha, 410205 Hunan China; 2 School of Medicine, Jinan University, Guangzhou, 510632 Guangdong China; 3 Biomedical Translational Research Institute, The First Affiliated Hospital, Jinan University, 601 W. Huangpu Ave., Guangzhou, 510632 Guangdong China

**Keywords:** Cancer genomics, Tumour biomarkers

## Abstract

SLMO2 is a lipid transporter that transports phosphatidylserine to the interior of mitochondria, also known as PRELID3B, which plays an important role in lipid metabolism. It has also been reported to be involved in the growth process of breast and lung tumors. However, its functions and underlying mechanisms in cancer progress remain elusive, and the potential as pan-cancer biomarker and therapeutic target remains unexplored. Using the TCGA project and GEO database, we performed pan-cancer analysis of SLMO2, which including the expression pattern, prognostic value, mutation landscape, methylation modification, protein–protein interaction network and the relationship between SLMO2 expression and immune infiltration. KEGG enrichment analysis was also performed to predict function and relevant cellular pathways of SLMO2. In addition, proliferation and migration assays were performed to detect the proliferation and metastasis capacity of breast cancer and lung cancer cells. In our study, we found that SLMO2 was overexpressed in pan-cancer and the elevated expression of SLMO2 was correlated with poorer prognosis. *SLMO2* mutations were distributed in a variety of tumors and correlated with prognosis. Promoter methylation analysis showed that *SLMO2* methylation levels were lower in most tumors compared with normal tissues, while a few tumors showed increased methylation levels of *SLMO2*. SLMO2 expression was also positively correlated with immune infiltration of MDSCs. Further pathway enrichment analysis indicated that SLMO2 was involved in regulating of cytoplasmic transport and other oncogenic processes. In vitro experiments have shown that SLMO2 promotes the proliferation and migration of breast cancer and lung cancer cells. In conclusion, our findings suggested that SLMO2 was a potential prognostic and immunological marker in pan-cancer. This study suggested a potential strategy for targeting SLMO2 to treat tumors, including manipulating tumor growth or the tumor microenvironment, especially the infiltration of MDSC.

## Introduction

The *Slowmo (SLMO)* gene has proved to encode a novel mitochondrial protein and to be essential for the developing central nervous system^1^. Slowmo Homolog 2 (*SLMO2*), also referenced as PRELI Domain Containing 3B (*PRELID3B*), belongs to the Ups/PRELI family^2^. This gene family contains six members, including *PRELID1, PRELID2, PRELID3A, PRELID3B, SCE1L1 and SCE1L5*. SLMO proteins contain the conserved PRELI/MSF1p' domain and are present in proteins of various eukaryotes^3^. Another important family member is called *PRELID3A*. However, the function of this family of proteins continues being unknown. Current studies have shown that SLMO2-TRIAP1 can act as a lipid transfer protein in the phosphatidylserine (PS)—specific mitochondrial cytomembrane space, allowing Psd1 to form PS in the inner membrane, and SLMO2 may be related to the function of combining protein and lipid homeostasis to maintain mitochondrial structure and function^1^. In addition, it has been proven that SLMO2-ATP5E is differentially expressed in colorectal cancer tissues^4^. These studies suggest that SLMO2 participates in cellular activities and may also play some specific roles in tumor cells. Therefore, the pan-cancer database should be utilized for further study with the regulatory function and the molecular mechanism of SLMO2 in tumors, understand the role of SLMO2 in tumorigenesis and development, and provide new directions and strategies for the clinical treatment of cancer.

Admittedly, cancers has become an important cause of death threatening human health^5^. The incidence and mortality of cancers are increasing with each passing year worldwide, which seriously endanger public health. Although there has been another clinical success in cancer treatment, the prognosis and survival rate of patients is still unsatisfactory due to drug resistance, side effects, and other problems^6^. According to recent studies, tumor immunity plays an active role in tumor microenvironment (TME) in the occurrence, progression, recurrence and metastasis of tumors, so it is therefore imperative that further research be conducted^7–9^. At the same time, it can also provide new theoretical support for the discovery of novel cancer biomarkers for tumor diagnosis and new strategies for cancer treatment^10,11^.

In this research, we carried out a systematic bioinformatics analysis based upon the existing abundant cancer data to elucidate the expression patterns and biological functions of SLMO2 in pan-cancer in multiple dimensions. Our study deepened the understanding of the function of SLMO2 in tumorigenesis, highlighted the potential of SLMO2 as a pan-cancer prognostic and immunological biomarker, and explored the underlying mechanisms of SLMO2 in different cancers.

## Methods

### Gene and protein expression analysis

Tumor Immune Estimation Resource 2.0 (TIMER2.0, http://timer.cistrome.org/, accessed on 17 November 2022) is a comprehensive resource for systematized analysis of immune infiltrates across diverse cancer types^12^. We input “SLMO2” into TIMER2.0 web to examine the difference in SLMO2 expression both tumors and neighboring normal tissues about the 32 cancer types in data from The Cancer Genome Atlas (TCGA).

The Cancer Cell Line Encyclopedia (CCLE, https://sites.broadinstitute.org/ccle/, accessed on 15 September 2023) performs detailed genetic and pharmacological characterization of a large number of human cancer models^13,14^. We input "*PRELID3B*" in the "database" module to explore and analyze the expression of SLMO2 in tumors.

GEPIA2 (http://gepia2.cancer-pku.cn/, accessed on 25 December 2022) is a Web-based online analysis tool for analyzing the RNA sequencing expression data of 9,736 tumors and 8,587 normal samples^15^. Its functions include differential expression analysis, correlation analysis, and patient survival analysis. We analyzed the expression of SLMO2 across TCGA tumors.

UALCAN (http://ualcan.path.uab.edu, accessed on 11 March 2023) is a comprehensive, interactive web-based resource for analyzing cancer-omics data, which contains clinical data for 31 cancer types^16^. Hereon, SLMO2 expression levels were measured in tumor and normal samples, as well as in tumor models based on tumor stages.

### Immunohistochemistry staining

The Human Protein Atlas (HPA; http://www.proteinatlas.org/, accessed on 25 March 2023) was used to obtain IHC images of SLMO2 protein expression^17^.

### Analysis of subcellular localization

HPA (http://www.proteinatlas.org/, accessed on 25 March 2023) was used to obtain IFC images of the subcellular localization of SLMO2 protein^18^.

### Survival prognosis analysis

The Kaplan–Meier Plotter (http://kmplot.com/analysis/, accessed on 25 December 2022) is able to assess the correlation between the expression of all genes (mRNA, miRNA, protein) and survival in 30k + samples from 21 tumor types. Sources of the databases include GEO, EGA, and TCGA^19,20^. A correlation was found between SLMO2 expression and survival in different cancers.

The GEPIA2.0 (accessed on 19 March 2023) was used only for patient survival analysis based on the TCGA database. We are input SLMO2 gene into the "survival analysis" module, select relevant cancer types, and analyse overall survival. We divided high- and low-expression cohorts by 50% thresholds.

### Genetic alteration analysis

The cBioPortal web (https://www.cbioportal.org/, accessed on 24 March 2023) was used for genetic alteration analysis^21^. We used this database to perform a pan-cancer mutation frequency analysis of SLMO2.

### DNA methylation analysis

DNA methylation is one of the most common forms of epigenetic modification in tumor development. We retrieve the UALCAN database (http://ualcan.path.uab.edu/, accessed on 24 March 2023), to explore some SLMO2 promoter DNA methylation in cancer, to determine the difference between tumor and normal tissue. Using DNA methylation data, MethSurv performs multivariable survival analysis (https://biit.cs.ut.ee/methsurv/, accessed on 21 April 2023)^22^.

### Immune infiltration analysis

We used the TIMER2.0 (http://timer.cistrome.org/, accessed on 19 March 2023) provides immune infiltrates' abundance estimated by multiple immune deconvolution methods. To determine the correlation between its expression and immune infiltrates of different cancers, we injected the SLMO2 gene into the "Genes" module of the "Immune" section. Infiltrating immune cells were rated on a critical scale, including CD4 + T cells, CD8 + T cells, and so on.

### Gene enrichment analysis related to SLMO2

In STRING (https://string-db.org/, accessed on 25 December 2022), we include both direct (physical) and indirect (functional) interactions between proteins. We employed STRING to obtain SlMO2-binding proteins and performed functional enrichment analysis of protein–protein interaction network^23^.

TIMER2.0 (accessed on 20 March 2023) was used to investigate the correlation between SLMO2 and a list of 20 indicators of SLMO2-binding in various cancer types. We input the *slmo2* and a list of genes of the 18 indicators (MZT2B and NELFCD is not retrieved in the database) into the “Gene_Corr” section.

Jvenn (http://www.bioinformatics.com.cn/static/others/jvenn/) is an interactive online tool for comparing lists and produced Venn diagrams^24^. We pasted the lists into the corresponding elements and performed an intersection analysis to compare the genes that bound to SLMO2 and those that interacted.

ShinyGO (http://bioinformatics.sdstate.edu/go/), a gene enrichment tool set^25,26^, combining 100 *SLMO2*-related genes in GEPIA.

### Cell lines

Breast Carcinoma Cells (MDA-MB-231) and Non-Small Cell Lung Cancer Cells (A549) cells were purchased from ATCC and cultured in Dulbecco’s modified Eagle’s medium (DMEM) supplemented with 10% heatinactivated fetal bovine serum (FBS) at 37 °C in a 5% CO_2_/95% air incubator.

### Antibodies

Anti-PRELID3B (SLMO2) was obtained from SAB Signalway. Anti-β-actin were obtained from Proteintech.

### Western blot

Cells were lysed with lysis buffer for 30 min on ice. After centrifugation, proteins were boiled in loading buffer for six minutes and fractionated by SDS-PAGE.

### Transfections

MDA-MB231 and A549 cells cultured in Dulbecco’s modified Eagle’s medium (DMEM) supplemented with 10% fetal bovine serum (FBS) were transfected with nontargeting control siRNAs and siRNA-SLMO2. Cells were harvested 48h after reverse transfection with Lipofectamine 2000 (Invitrogen).

### Cell proliferation assay

MDA-MB-231 and A549 cells (2 × 10^5^) were inoculated on a 6-well plate and cultured for 3 days. The number of cells was counted at 24, 48 and 72 h, respectively, and the cell proliferation rate was calculated.

### Clone formation assay

MDA-MB-231 cells and A549 cells (100 cells) were placed in 6-well plates and cultured at 37 °C for 14 days until the lesions were obvious. The colonies were fixed with 4% formaldehyde and stained with 0.5% crystal violet. After staining, the colonies were washed with PBS and counted.

### Transwell assay for migration

MDA-MB-231 and A549 cells (5 × 10^4^ cells) were re-suspended in serum-free medium, inoculated (for migration) in transwell inserts, and then cultured for 16 h. Cells infiltrated into the lower surface of transwell inserts were immobilized with methanol, stained with 0.5% crystal violet, and counted.

### Statistical analysis

Data from biological triplicate experiments were presented with error bar as mean ± SD. Two-tailed unpaired Student's t-test was used for comparing two groups of data. Statistical significance was determined by *p*-values less than 0.05. The following annotations were used to illustrate significance: *p < 0.05, **p < 0.01, ***p < 0.001, and ****p < 0.0001.

## Results

### mRNA expression of *SLMO2* in human pan-cancer

**Figure 1:**
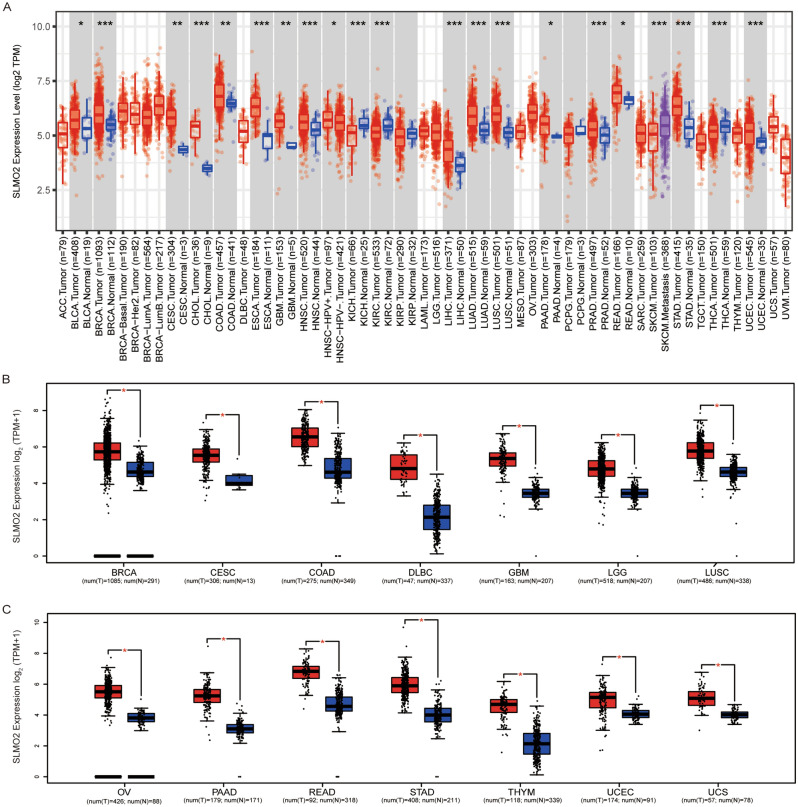
mRNA Expression of SLMO2 in Human Pan-Cancer. (**A**) mRNA expression levels of SLMO2 were analyzed in different cancer types from TCGA data in TIMER2.0. *p < 0.055, **p < 0.01, ***p < 0.001. (**B**,**C**) Differences of SLMO2 expression between cancers from the TCGA database and normal samples from the GTEx database. Box plot data were supplied. *p < 0.05.

Based on data from TCGA and GTEx databases, we evaluated the expression of *SLMO2* mRNA in tumor and normal tissues. To investigate the differences in *SLMO2* expression between tumors and adjacent normal tissues, we analyzed *SLMO2* mRNA expression levels in all TCGA tumors through TIMER2.0 databases. The results showed that *SLMO2* was highly expressed in BLCA (bladder urothelial carcinoma), BRCA (breast invasive carcinoma), CESC (cervical squamous cell carcinoma and endo-cervical adenocarcinoma), CHOL (cholangiocarcinoma), COAD (colon adenocarcinoma), ESCA (esophageal carcinoma), GBM (glioblastoma multiforme), HNSC (head and neck squamous cell carcinoma), LIHC (liver hepatocellular carcinoma), LUAD (lung adenocarcinoma), LUSC (lung squamous cell), PAAD (Pancreatic adenocarcinoma), PRAD (prostate adenocarcinoma), READ (rectum adenocarcinoma), STAD (stomach adenocarcinoma), and UCEC (uterine corpus endometrial carcinoma) compared with their adjacent normal tissues (Fig. 1A). We further used the GEPIA2 database to analyze the expression of SLMO2 in pan-cancer, which incorporated GTEx dataset into normal samples to expand the number of normal samples. Compared with normal tissues, 14 cancer types expressed higher levels of SLMO2 (Fig. 1B,C). In order to verify the accuracy of the results, we further analyzed the data of the Cancer Cell Line Encyclopedia (CCLE) database. Consistent with previous data, the CCLE data further confirmed that SLMO2 was highly expressed in most tumors (Fig. S1A and 1B). According to all the data, SLMO2 was expressed highly in most cancers.

### Expression and subcellular localization of SLMO2 in cancers

**Figure 2:**
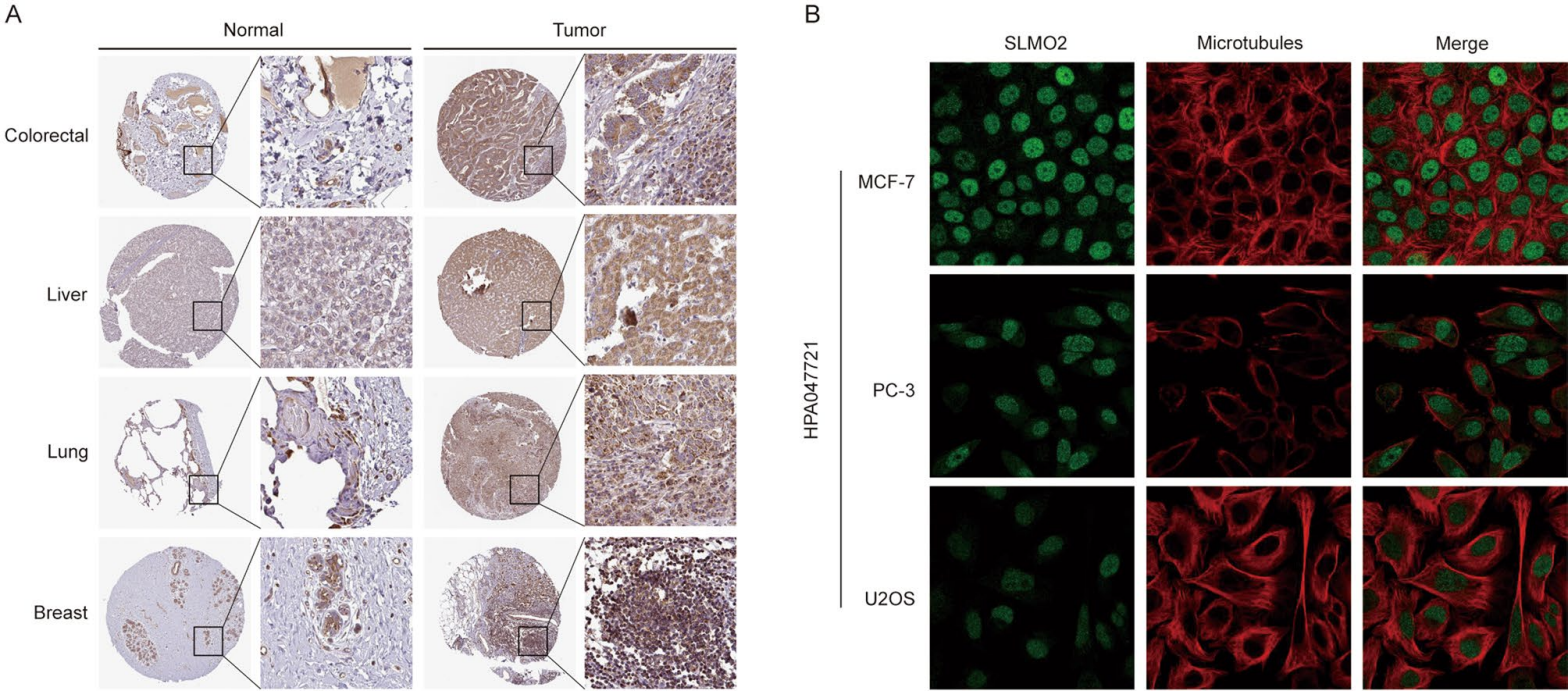
Expression and Subcellular Localization of SLMO2 in Cancers. (**A**) Protein expression of SLMO2 in BRCA, COAD, LIHC and LUCA tissues from the HPA database. (**B**) Immunofluorescence staining of the subcellular localization of SLMO2 were analyzed in MCF-7, PC-3 and U2SO from the HPA database.

Proteins are the principal molecules that are most directly related to diseases, and changes in protein expression levels are directly related to diseases, drug effects or toxic effects. Using the Human Protein Atlas (HPA) datasets, we further investigated the differential expression of SLMO2 between tumors and normal tissues by immunohistochemistry. SLMO2 expression was significantly increased in BRCA, COAD, LIHC and LUCA tissues compared to normal tissues (Fig. 2A). We also found that SLMO2 was localized in the nucleus of MCF-7, PC-3 and U2SO cells (Fig. 2B).

### High expression of SLMO2 in pan-cancer on different stages

**Figure 3:**
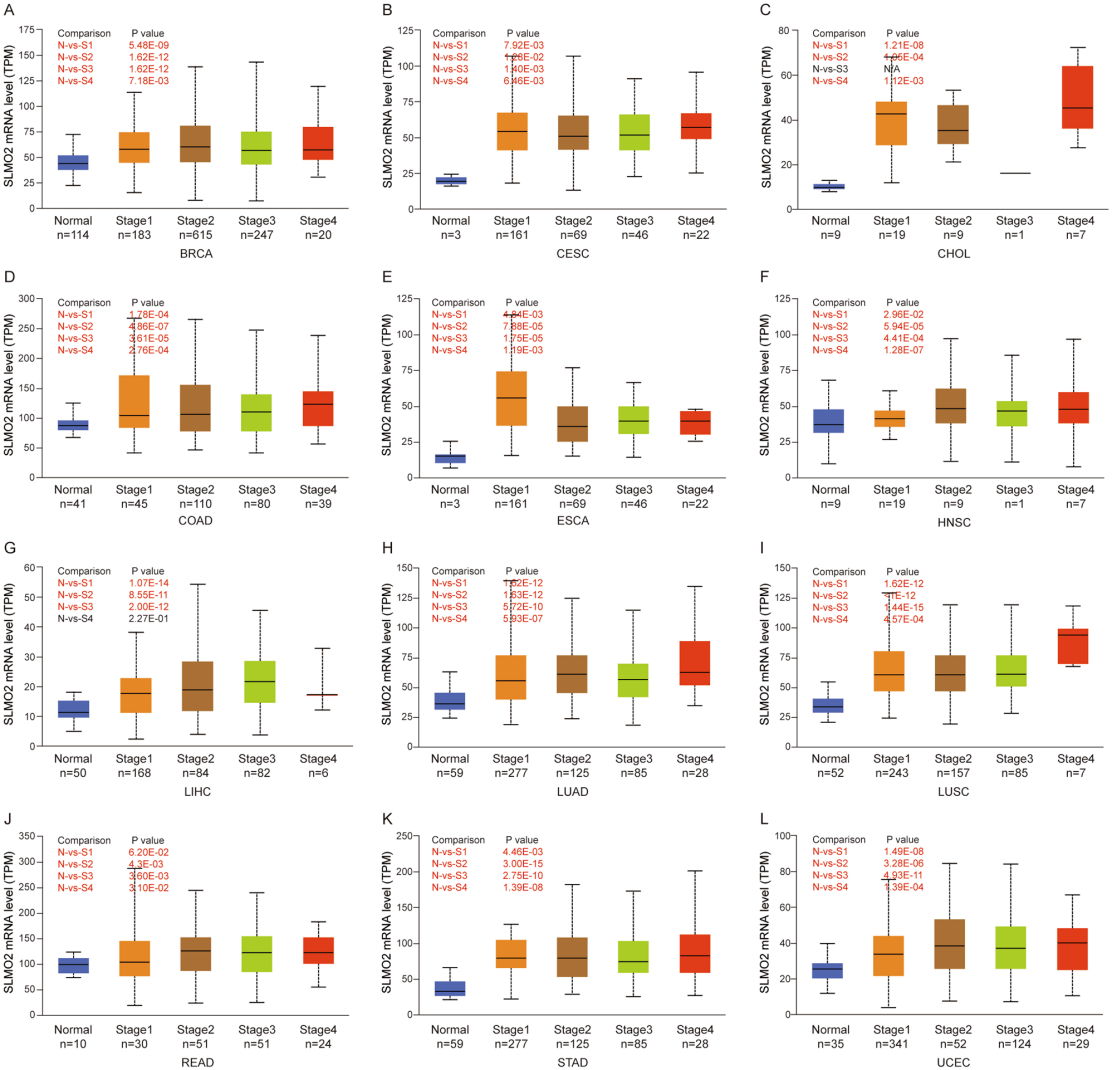
High Expression of SLMO2 in Pan-Cancer on Different Stages. The expression of SLMO2 according to the pathological stage of the patient in the TCGA cancer type in UALCAN database. X axis: pathological cancer stages with the number of samples in each stage. Y axis: transcript per million. N: normal. S: stage. *p*-Value marked red means the two groups are statistically significant.

The expression of SLMO2 was examined according to the pathological stage of patients with TCGA cancer types. The results showed that in BRCA, CESC, CHOL, COAD, ESCA, HNSC, LIHC, LUAD, LUSC, READ, STAD and UCEC, the expression level of SLMO2 was significantly higher in tumor stage compared with normal tissues. However, there were no significant differences between early stage and late stage (Fig. 3), which hinted that SLMO2 may be involved in tumor initiation but not cancer progression.

### High levels of *SLMO2* predicts poor clinical outcomes in several cancer types

**Figure 4:**
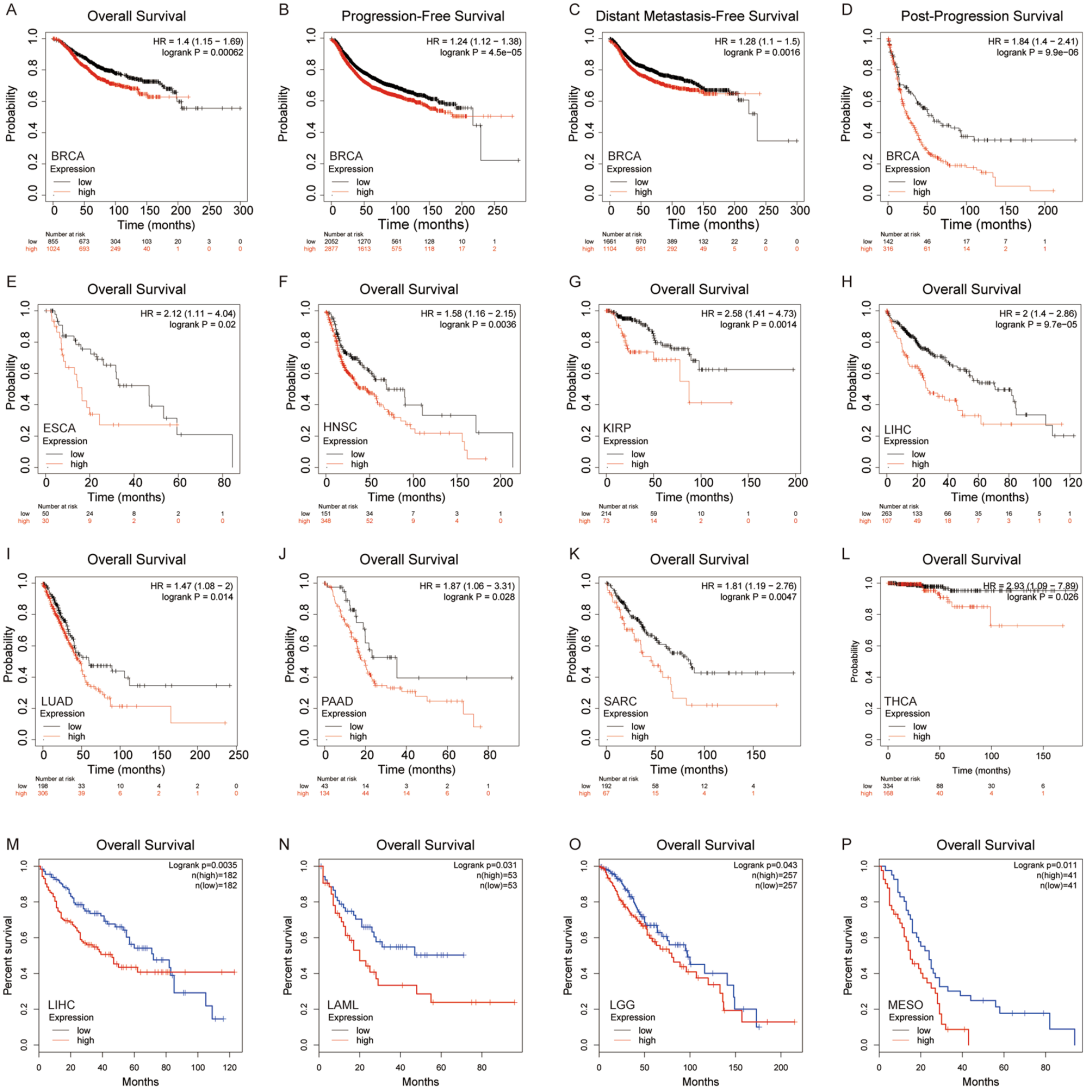
High Levels of SLMO2 Predicts Poor Clinical Outcomes in TCGA. (**A**–**D**) The Kaplan–Meier plotter tool analysis showed that SLMO2 expression was negatively correlated with different prognostic types in breast cancer patients. (**E**–**L**) The survival data for different tumors were analyzed using the Kaplan–Meier plotter tool. (**M**–**P**) In GEPIA2, the TCGA and GEO database was used to analyze the relationship between SLMO2 expression and survival prognosis of patients with different tumors.

Based on the mRNA expression level of *SLMO2*, we divided the patients into high and low groups, then evaluated the correlation between *SLMO2* expression and prognosis in different tumors. The results showed high expression of *SLMO2* was associated with poorer survival (OS) overall survival (OS) in BRCA (*p* = 0.00062) (Fig. 4A). In addition, we also found that high expression of *SLMO2* was associated with poor prognosis of progression-free survival (PFS) (*p* = 4.5e−05), distant metastasis-free survival (DMFS) (*p* = 0.0016) and post-progressive survival (PPS) (*p* = 9.9e−06) in BRCA (Fig. 4B–D). *SLMO2* expression was closely associated with poor prognosis in BRCA, which may serve as a prognostic indicator.

In addition, the prognostic value of SLMO2 in other tumors was further investigated through the TCGA database pair of Kaplan–Meier plotter and GEPIA. Compared to low expression levels, consistent with BRCA, Kaplan–Meier plotter showed that a high expression level of SLMO2 was correlated with a worse OS in ESCA (*p* = 0.02), HNSC (*p* = 0.0036), KIRP (*p* = 0.0014), LIHC (*p* = 9.7e−05), LUAD (*p* = 0.014), PAAD (*p* = 0.028), SARC (*p* = 0.0047), THCA (*p* = 0.026) (Fig. 4E–L). Based on the GEPIA dataset, we verified that SLMO2 expression had a poor prognosis of OS in LIHC (*p* = 0.0035), LAML (*p* = 0.031), LGG (*p* = 0.043), MESO (*p* = 0.011) (Fig. 4M–P). The above data indicated that high expression of SLMO2 was closely associated with poor prognosis in various cancers, suggesting that SLMO2 was a promising pan-cancer prognostic biomarker.

### Mutation feature of *SLMO2* in pan-cancer

**Figure 5:**
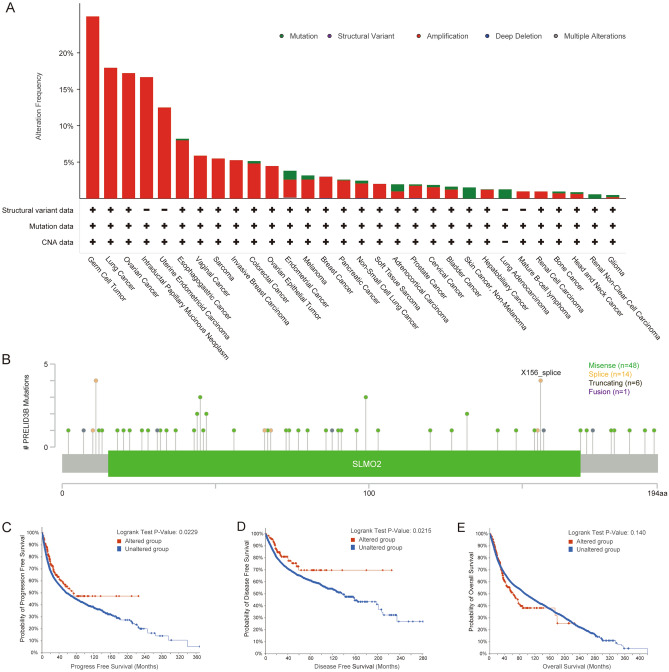
Mutation Feature of SLMO2 in Pan-Cancer. (**A**) Mutation types and alteration frequencies of SLMO2 in different tumors. (**B**) SLMO2 gene mutation sites and the number of cases. (**C**,**D**) The association between SLMO2 genetic alterations and clinical survival prognosis.

To elucidate the mutational signature and biological function of *SLMO2* in tumor progression, we discussed *SLMO2* genetic alterations in pan-cancer using the cBioPortal database. The results showed that lung cancer carried the highest frequency of *SLMO2* mutations (17.95%), mainly manifested as "Amplification". Additionally, high frequencies of *SLMO2* mutations were found in OV (17.22%) and UEC (12.5%), predominantly in the "Amplified" form (Fig. 5A). Furthermore, we assessed the types, sites, and case numbers of *SLMO2* gene alterations. We found that missense mutations with *SLMO2* were detected in 48 cases and were the predominant type of genetic alteration. In addition, 6 cases contained truncating mutation and 14 cases spliced mutation. Fusion was detected in only one case (Fig. 5B). In addition, we explored the association between *SLMO2* genetic alterations and clinical survival in pan-cancer. Surprisingly, we found *SLMO2* genetic alterations were associated with prolonged PFS and DFS (Fig. 5C,D), which suggested that *SLMO2* mutations may prematurely terminate protein synthesis and inhibit its function. However, there was no significant change in the OS (Fig. 5E), which suggested that there may be a genetic compensation effect in the cells, but further exploration is needed.

### DNA methylation level of *SLMO2* in pan-cancer

**Figure 6:**
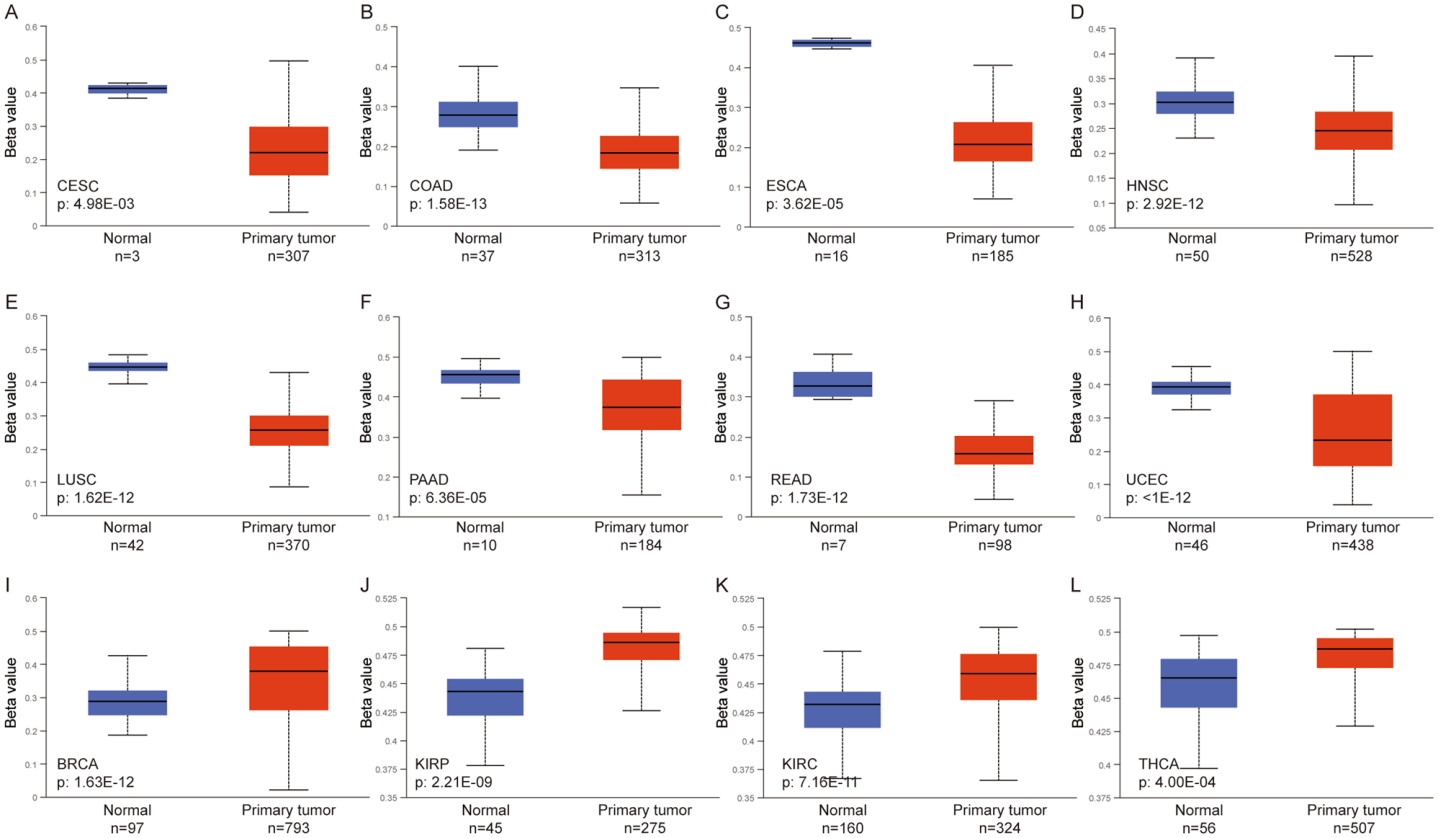
DNA Methylation Level of SLMO2 in Pan-Cancer. (**A**–**L**) Promoter methylation level of SLMO2 in CESC, COAD, ESCA, HNSC, LUSC, PAAD, READ, UCEC, BRCA, KIRP, KIRC and THCA.

Aberrant DNA methylation control mechanisms lead to a variety of diseases, including cancer^27^. Cancer cells are characterized by aberrant DNA methylation, including genomic hypomethylation and site-specific hypermethylation^27,28^. We examined the DNA methylation level of *SLMO2* in various tumors using UALCAN database. The results showed that the methylation level of *SLMO2* in CESC, COAD, ESCA, HNSC, LUSC, PAAD, READ and UCEC was lower than that in normal tissues (Fig. 6A–H). This may be an explanation for the high expression of *SLMO2* in these tumors. In BRCA, KIRP, KIRC and THCA, the methylation level of *SLMO2* was higher than that of normal tissues (Fig. 6I–L).

MethSurv was used to analyze the correlation between DNA methylation level of *SLMO2* and survival rate. We analyzed nine methylation probes associated with *SLMO2* in the MethSurv database, including: cg02912129, cg03255221, cg06943251, cg08363339, cg12102151, cg14073986, cg20623172, cg20726575, and cg26216876 (Supplementary Table S1). Additionally, we examined the correlation between *SLMO2* DNA methylation and prognosis in different tumors. The results showed that, the prognosis of hypermethylation was benefit in LIHC, ESCA, PAAD, LUSC, UCEC, LGG, ACC, LUAD, and GBM (Supplementary Fig. S1). *SLMO2* is affected by methylation or demethylation modification, which leads to the change of expression level and plays a carcinogenic role, and may affect the prognosis of cancer patients through methylation.

### Correlation analysis between SLMO2 expression and immune infiltration of MDSC

**Figure 7:**
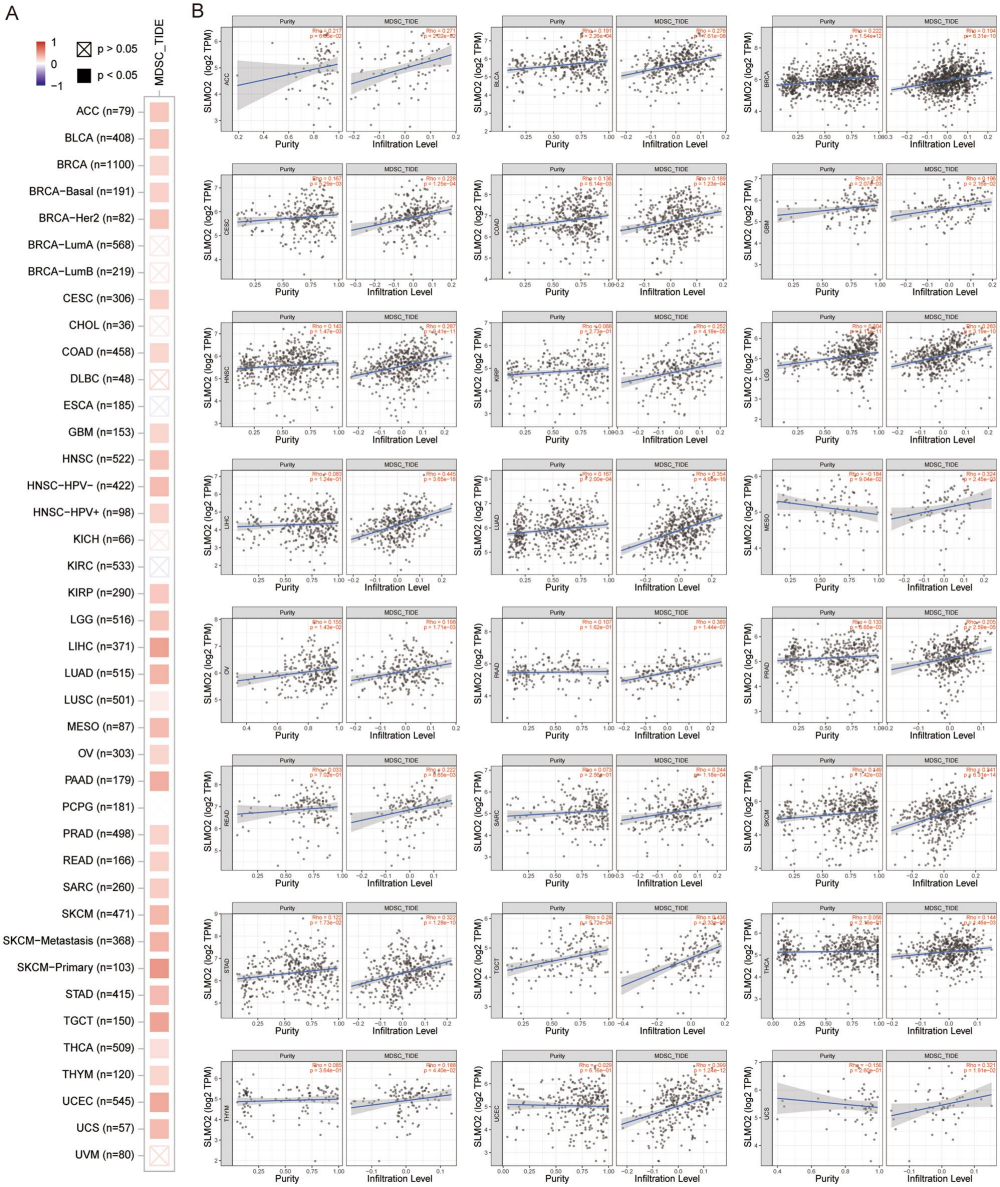
Correlation Analysis between SLMO2 Expression and Immune Infiltration of MDSC. (**A**) The relationship between MDSC infiltration and SLMO2 gene expression was shown by heatmap in TCGA across all cancer types. (**B**) Correlation between MDSC infiltration and SLMO2 gene expression was exhibited by scatter plot in TCGA.

The level of immune infiltration in the tumor microenvironment (TME) is closely related to cancer occurrence, progression, and spread^9^. Using the TIMER2.0 database, we investigated the correlation between SLMO2 expression levels and the infiltration of various immune cell subsets in the tumor microenvironment. We found that the expression level of SLMO2 was positively correlated with the infiltration level of MDSCs in most cancer types (Fig. 7A,B), but not with the infiltration of other immune cell subsets, including B cells, CD4+ T cells, CD8+ T cells, myeloid cells, macrophages, NK cells, Tfh cells, γδ T cells, Tregs, monocytes and neutrophils (Supplementary Fig. S2 and S3). Furthermore, we investigated the correlation between MDSC infiltration level and prognosis in the TCGA dataset of TIMER2.0. The results showed that in most tumors, high levels of MDSC infiltration were associated with a poor prognosis (Fig. S4). Our studies indicated that SLMO2 was involved in the tumor immunology process.

### SLMO2 related gene enrichment analysis

**Figure 8:**
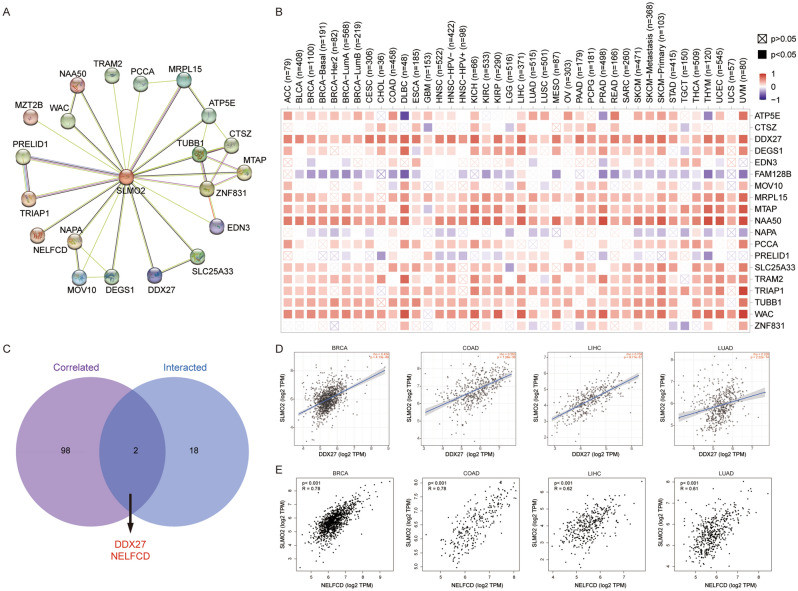
SLMO2 Related Gene Enrichment Analysis. (**A**) Prediction analysis of SLMO2 interacting proteins. (**B**) Correlation of SLMO2 with 20 interacting proteins bound by SLMO2 in pan-cancer. (**C**) Intersection analysis of SLMO2-related genes and SLMO2-interaction partners. (**D**) Correlations of SLMO2 with DDX27 and NELFCD.

SLMO2-binding proteins were screened using protein–protein interaction network analysis to further understand the molecular mechanism of SLMO2. STRING online tool provided 20 SLMO2-binding proteins, including those supported or predicted by experimental evidence (Fig. 8A). Then the expression correlation of SLMO2 with 20 SLMO2 PPI members was further analyzed. And we found the DDX27, DEGS1, MRPL15, MTAP, NAA50, TRAM2, TRIAP1, TUBB1, WAC and SLMO2 present obvious positive correlation in most tumors (Fig. 8B). Then, the GEPIA2 tool was utilized for combining all tumor expression data from TCGA to obtain the top 100 genes associated with SLMO2 expression. DDX27 and NELFCD were found to have two common members by intersection analysis (Fig. 8C). Further correlation analysis indicated that DDX27 and NELFCD were strongly positively correlated with SLMO2 expression (Fig. 8D,E). In addition, we used the ShinyGO tool for enrichment analysis. Here, we performed the Kyoto Encyclopedia of Genes and Genomes (KEGG) pathway enrichment analysis, we found that the SLMO2 was involved in the "Nucleocytoplasmic transport" and "Endocytosis" pathway (Table 1).

**Table 1:** SLMO2 related gene enrichment analysis.

*p*-Value	nGenes	Pathway genes	Fold enrichment	Pathway	Genes
0.002	6	108	11.61875637	Nucleocytoplasmic transport	XPO1 RAE1 KPNB1 NUP155 CSE1L XPOT
0.088	6	252	4.979467016	Endocytosis	ITCH SNX4 ARFGEF2 RAB22A STAM ASAP2

### SLMO2 promotes proliferation and migration of breast cancer and lung cancer cells in vitro

**Figure 9:**
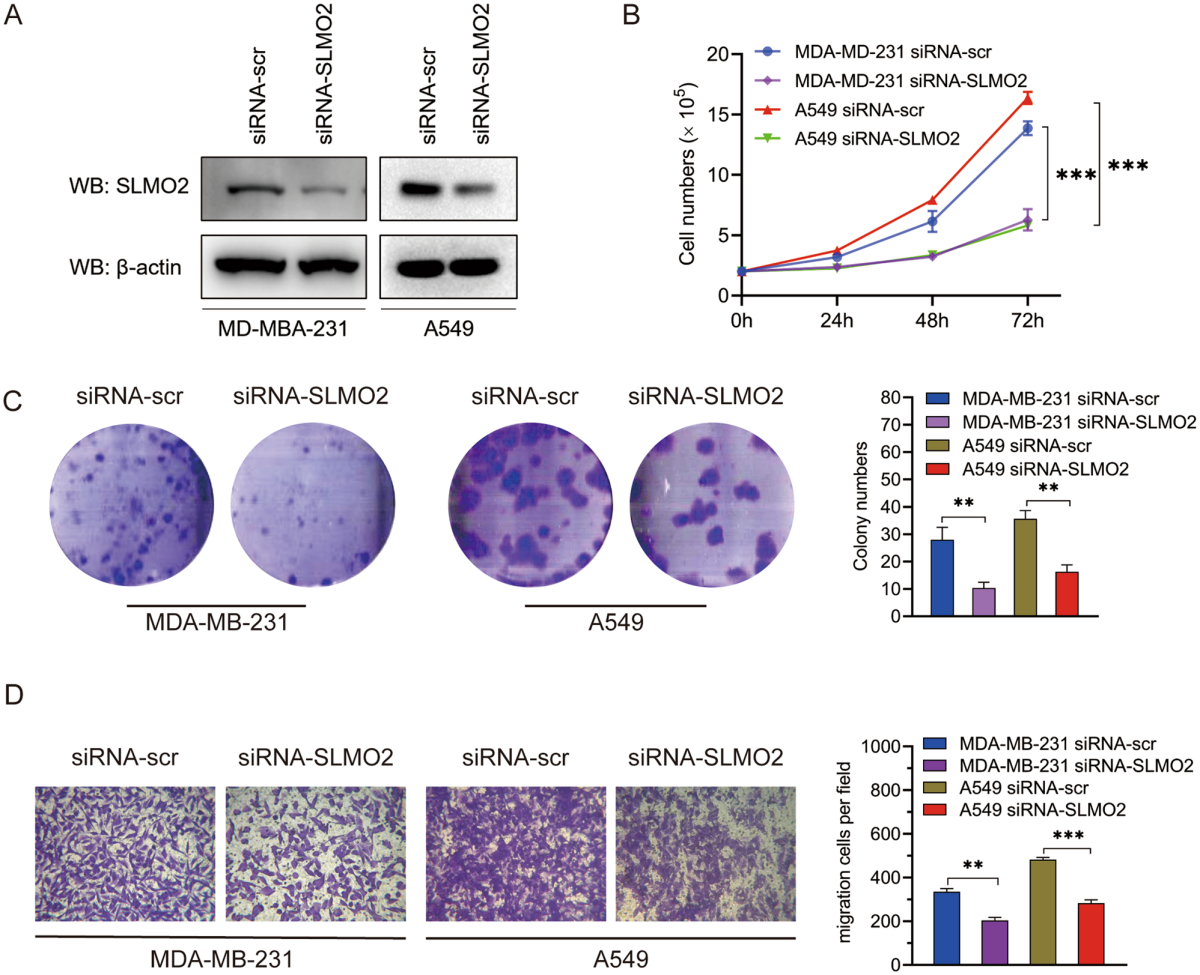
Effects of SLMO2 on proliferation and migration of MDA-MB-231 and A549 cells in vitro. (**A**) Western blot was used to detect siRNA-SLMO2 infection in MDA-MB-231 and A549 cells. (**B**) Proliferation assay was used to evaluate cell proliferation in MDA-MB-231 and A549 cells. (**C**) Clone formation assay was performed to evaluate the growth of MDA-MB-231 and A549 cells. (**D**) Transwell assays were performed to assess cell migration in MDA-MB-231 and A549 cells.

Considering the key role of SLMO2 regulation and the data analysis of SLMO2 in breast cancer and lung cancer growth and metastasis, we speculate that SLMO2 may play a role as an oncoprotein in breast cancer and lung cancer cells. To test this hypothesis, we first examined the protein expression level of SLMO2, and the results showed that SLMO2 protein expression was significantly down-regulated in SLMO2 knockdown (siRNA-SLMO2) MDA-MB-231 and A549 cells (Fig. 9A).

Then, we performed proliferation assays using siRNA scrambled control (siRNA-scr) and SLMO2 knockdown (siRNA-SLMO2) MDA-MB-231 and A549 cells. Our results showed that the ablation of SLMO2 by siRNA significantly impaired the proliferation of MDA-MB-231 and A549 cells (Fig. 9B). Next, we implemented clonogenic assays. The colony numbers were markedly decreased by siRNA-SLMO2 MDA-MB-231 and A549 cells (Fig. 9C). These findings suggest that SLMO2 maintains the proliferation and survival of breast cancer and lung cancer cells. SLMO2 plays a key role in breast cancer and lung cancer metastasis. We determined the effect of SLMO2 on MDA-MB-231 and A549 cells migration by transwell assay. As expected, SLMO2 knockdown significantly reduced the ability of MDA-MB-231 and A549 cells to migrate through the transwell membrane (Fig. 9D). Taken together, these findings suggest that SLMO2 promotes the growth and migration on breast cancer and lung cancer cells.

## Discussion

As a novel mitochondria-associated protein, previous reports only showed that *SLMO2* was associated with lipid transport and protein stabilization, and was differentially expressed in colorectal cancer^1^. However, no reports have in fact demonstrated the correlation between SLMO2 and tumor progression. Therefore, we investigated the functional expression of SLMO2 as well as its prognostic role in multiple tumors by systematic pan-cancer analysis. This analysis included the effect of *SLMO2* RNA expression level on prognosis, genetic alterations of *SLMO2* genes, tumor immunity of *SLMO2* related genes, and KEGG analysis^26^.

In this study, we identified that SLMO2 was significantly up-regulated in BLCA, BRCA, CESC, CHOL, COAD, ESCA, GBM, HNSC, LIHC, LUAD, LUSC, PAAD, PRAD, READ, STAD, and UCEC. And this high expression can lead to a worse clinical prognosis of patients. In addition, we also found that the expression of SLMO2 was higher in different stages of tumor than that in the control group, while there was no significant difference in the expression during different stages, suggesting that the main role of SLMO2 in tumor is to promote tumorigenesis, and the development of the tumor needs further investigation.

Genetic alterations, as an important influence on tumorigenesis, also play an important role in SLMO2. The mutation profile of *SLMO2* pan-carcinoma showed that the frequency of *SLMO2* mutation was the highest in LUCA, reaching 17.95%. However, clinical survival analysis of pan-cancer showed that the survival rate of *SLMO2* altered tumors was worse than that of the *SLMO2* unchanged group. We believe that this may be because alterations in *SLMO2* affect the transcriptional regulation of the *SLMO2* gene as well as the functional role after translation.

As one of the common epigenetic modifications, DNA methylation plays a crucial role in gene expression, transcriptional regulation and tumorigenesis. Studies have shown that aberrant DNA methylation can promote cell proliferation to accelerate tumor development^27,28^. Using the UALCAN, we observed that SLMO2 promoter methylation levels were significantly lower in CESC, COAD, ESCA, HNSC, LUSC, PAAD, READ and UCEC, while higher in BRCA, KIRP, KIRC and THCA compared to normal tissues. In our study, we found that *SLMO2* DNA methylation was down-regulated in multiple tumors, which resulted in poor patient prognosis. These findings suggest that *SLMO2* may promote tumorigenesis through DNA methylation.

As a momentous sign of the most malignant tumors, tumor immune infiltration is closely related to the occurrence, development and metastasis of cancer in TME^29,30^. MDSC is one of the important immune cells in tumor^31^. MDSC protects cancer from the patient's immune system and can also make tumors resistant to immunotherapy^32,33^. Additionally, MDSC has been shown to promote tumor progression by promoting tumor cell survival, angiogenesis, invasion, and metastasis^29^. In the present study, SLMO2 was positively correlated with MDSC infiltration. However, regulation of SLMO2 by MDSC in TME is an extremely complex issue. Our understanding of SLMO2's role in tumors will be enhanced by further studies on the effect of high expression in tumor tissues on immune infiltration.

Protein–protein interaction (PPI) and correlation analysis of SLMO2 showed that DDX27 and NELFCD were positively correlated with SLMO2 expression. The DEAD-box RNA helicase DDX27 has been demonstrated to have oncogenic properties^34,35^. DDX27 promotes CRC progression by forming the DDX27-NPM1-NFκb axis^36^. The interaction between DDX27 and SLMO2 may also accelerate tumorigenesis. Similarly, it has also been proven that NELFCD, as a transcription factor, is up-regulated in colorectal cancer tissues and plays a carcinogenic role^37,38^. Whether the interaction between NELFCD and SLMO2 will play a synergistic role, participate in the nuclear and cytoplasmic transport functions, and jointly accelerate the development process.

Breast cancer and lung cancer are the most common cancers all around the world, and breast cancer has become one of the leading causes of death among women worldwide^39,40^. Further functional experiments showed that downregulation of *slmo2* expression significantly affected the proliferation and migration of breast cancer and lung cancer cells. Although we compared protein expression in breast cancer and lung cancer cells with normal breast and lung cancer cells through online database analysis and experiments, further evidence is needed to determine the mechanisms associated with SLMO2 affecting functional changes in breast cancer cells and even other cancers.

In this study, to sum up, the first study of SLMO2 was conducted in pan-cancer, including expression, survival prognosis, epigenetics, methylation, immunoassay and enrichment analysis. The up-regulation of SLMO2 affects pan-cancer prognosis and is inextricably linked to immune infiltration. The increased SLMO2 expression is combined with poor prognosis and increased immune infiltration level of MDSC. SLMO2 can be used as a pan-cancer prognostic biomarker, and we provide rationale and fundamental support for anti-tumor strategies targeting SLMO2.

Although our study systematically analyzed the role of SLMO2 in tumors, nevertheless, this study has certain limitations, such as the lack of systematic experimental validation, and it is worthwhile to further investigate the detailed carcinogenic mechanism of SLMO2 in pan-cancer or individual cancer through in vitro and in vivo experiments.

## Conclusions

In conclusion, our results showed that SLMO2 expression was significantly up-regulated in tumors and was closely related to immune infiltration and tumors’ proliferation and migration, providing research basis and support for SLMO2 regarding as a potential therapeutic and prognostic biomarker for cancers.

### Supplementary Information

Supplementary Information 1.

Supplementary Information 2.

## Data Availability

All the datasets analyzed in this study can be furtherly inquired here: TIMER2.0, http://timer.cistrome.org/; CCLE, https://sites.broadinstitute.org/ccle/; GEPIA2, http://gepia2.cancer-pku.cn/; Kaplan–Meier Plotter, http://kmplot.com/analysis/; UALCAN, http://ualcan.path.uab.edu; HPA, http://www.proteinatlas.org/; cBioPortal, https://www.cbioportal.org/; CPTAC: http://ualcan.path.uab.edu/; MethSurv, https://biit.cs.ut.ee/methsurv/; STRING, https://string-db.org/; Jvenn, http://www.bioinformatics.com.cn/static/others/jvenn/; ShinyGO, http://bioinformatics.sdstate.edu/go/.

## Abbreviations

TCGA: The cancer genome atlas
CPTAC: Clinical proteomic tumor analysis consortium
KEGG: Kyoto encyclopedia of genes and genomes
BLCA: Bladder urothelial carcinoma
BRCA: Breast invasive carcinoma
CESC: Cervical squamous cell carcinoma and endo-cervical adenocarcinoma
CHOL: Cholangio carcinoma
COAD: Colon adenocarcinoma
ESCA: Esophageal carcinoma
GBM: Glioblastoma multiforme
HNSC: Head and neck squamous cell carcinoma
KICH: Kidney chromophobe
KIRC: Kidney renal clear cell carcinoma
KIRP: Kidney renal papillary cell carcinoma
LIHC: Liver hepatocellular carcinoma
LUAD: Lung adenocarcinoma
LUSC: Lung squamous cell
PRAD: Prostate adenocarcinoma
READ: Rectum adenocarcinoma
STAD: Stomach adenocarcinoma
THCA: Thyroid carcinoma
UCEC: Uterine corpus endometrial carcinoma
BLCA: Bladder urothelial carcinoma
DLBC: Lymphoid neoplasm diffuse large B-cell lymphoma
GBM: Glioblastoma multiforme
LGG: Brain lower grade glioma
OV: Ovarian serous cystadenocarcinoma
UCS: Uterine carcinosarcoma
ccRCC: Clear cell renal cell carcinoma
OS: Overall survival
DFS: Disease-free survival prognosis
PFS: Progression-free survival
DMFS: Distant metastasis-free survival
Treg: Regulation T cell
Tfh: Follicular helper T cell
MDSC: Myeloid derived suppressor cell
PPS: Post-progressive survival
DDX27: DEAD-box helicase 27
NELFCD: Negative elongation factor proteins C and D
