# Author Correction: SLMO2 is a potential prognostic and immunological biomarker in human pan-cancer

**DOI:** 10.1038/s41598-024-55384-z

**Published:** 2024-03-13

**Authors:** Xiong Liu, Renming Yuan, Jie Peng, Ailei Xu, Xiaoxia Nie, Ruiti Tang, Guangqiang Li

**Affiliations:** 1 Clinical Laboratory, Hunan Aerospace Hospital, 189 Fenglin 3rd Road, Yuelu District, Changsha, 410205 Hunan China; 2 School of Medicine, Jinan University, Guangzhou, 510632 Guangdong China; 3 Biomedical Translational Research Institute, The First Affiliated Hospital, Jinan University, 601 W. Huangpu Ave., Guangzhou, 510632 Guangdong China

Correction to: *Scientifc Reports* 10.1038/s41598-024-51720-5, published online 11 January 2024

The original version of this Article contained errors in the Results, Discussion and Figures 6, 8, 9.

In the Results section, the subheading ‘High levels of *SLMO2* predicts poor clinical outcomes in TCGA’,

now reads:

“High levels of *SLMO2* predicts poor clinical outcomes in several cancer types”

Under the subheading, ‘Mutation feature of *SLMO2* in pan-cancer’,

“The results showed that LUCA carried the highest frequency of *SLMO2* mutations (17.95%), mainly manifested as “Amplifcation”.”

now reads:

“The results showed that lung cancer carried the highest frequency of *SLMO2* mutations (17.95%), mainly manifested as “Amplifcation”.”

“Additionally, high frequencies of *SLMO2* mutations were found in OV (17.22%) and UECA (12.5%), predominantly in the “Amplifed” form (Fig. 5A).”

now reads:

“Additionally, high frequencies of *SLMO2* mutations were found in OV (17.22%) and UEC (12.5%), predominantly in the “Amplifed” form (Fig. 5A).”

Under the subheading ‘DNA methylation level of *SLMO2* in pan-cancer’,

“The results showed that the methylation level of *SLMO2* in CESE, COAD, ESCA, HNSC, LUSC, PAAD, READ and UCEC was lower than that in normal tissues (Fig. 6A–H). This may be an explanation for the high expression of *SLMO2* in these tumors. In BRCA, KIPC, KIRC and THCA, the methylation level of *SLMO2* was higher than that of normal tissues (Fig. 6I–L).”

now reads:

“The results showed that the methylation level of *SLMO2* in CESC, COAD, ESCA, HNSC, LUSC, PAAD, READ and UCEC was lower than that in normal tissues (Fig. 6A–H). This may be an explanation for the high expression of *SLMO2* in these tumors. In BRCA, KIRP, KIRC and THCA, the methylation level of *SLMO2* was higher than that of normal tissues (Fig. 6I–L).”

In the Discussion section,

“Using the UALCAN, we observed that SLMO2 promoter methylation levels were significantly lower in CESE, COAD, ESCA, HNSC, LUSC, PAAD, READ and UCEC, while higher in BRCA, KIPC, KIRC and THCA compared to normal tissues.”

now reads:

“Using the UALCAN, we observed that SLMO2 promoter methylation levels were significantly lower in CESC, COAD, ESCA, HNSC, LUSC, PAAD, READ and UCEC, while higher in BRCA, KIRP, KIRC and THCA compared to normal tissues.”

In addition, several labels and tumor abbreviations in Figures 6, 8, 9 were incorrect. The original Figures 6, 8 and 9 and accompanying legends appear below.

**Figure 6:**
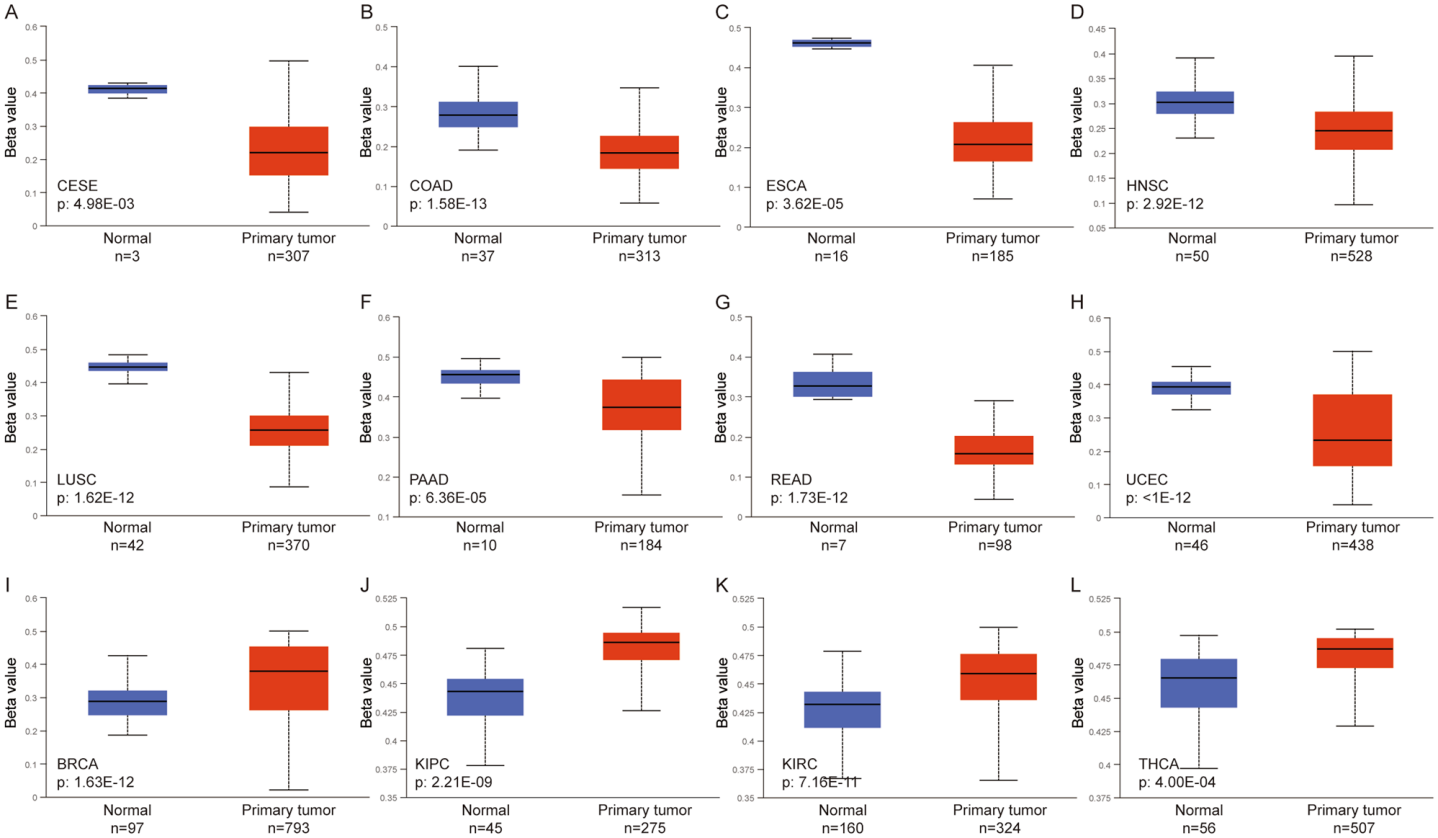
DNA Methylation Level of SLMO2 in Pan-Cancer. (**A**–**L**) Promoter methylation level of SLMO2 in CESE, COAD, ESCA, HNSC, LUSC, PAAD, READ, UCEC, BRCA, KIPC, KIRC and THCA.

**Figure 8:**
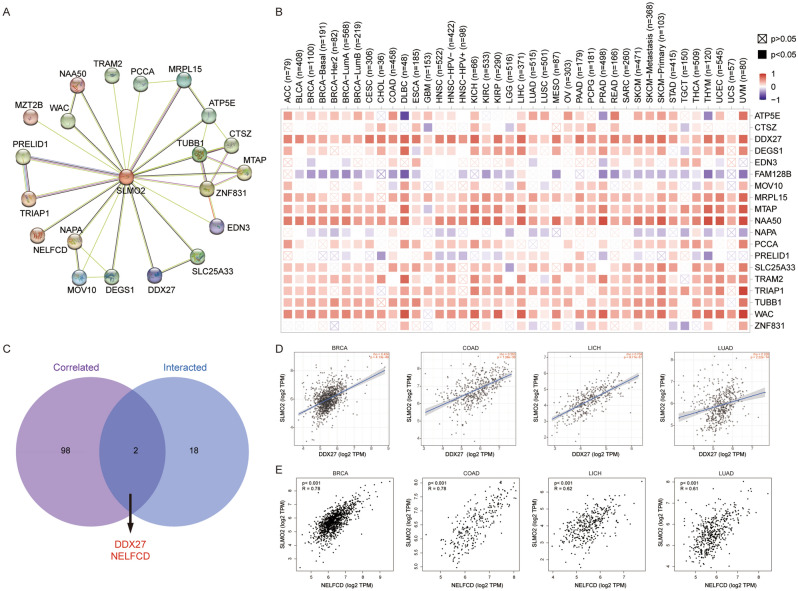
SLMO2 Related Gene Enrichment Analysis. (**A**) Prediction analysis of SLMO2 interacting proteins. (**B**) Correlation of SLMO2 with 20 interacting proteins bound by SLMO2 in pan-cancer. (**C**) Intersection analysis of SLMO2-related genes and SLMO2-interaction partners. (**D**) Correlations of SLMO2 with DDX27 and NELFCD.

**Figure 9:**
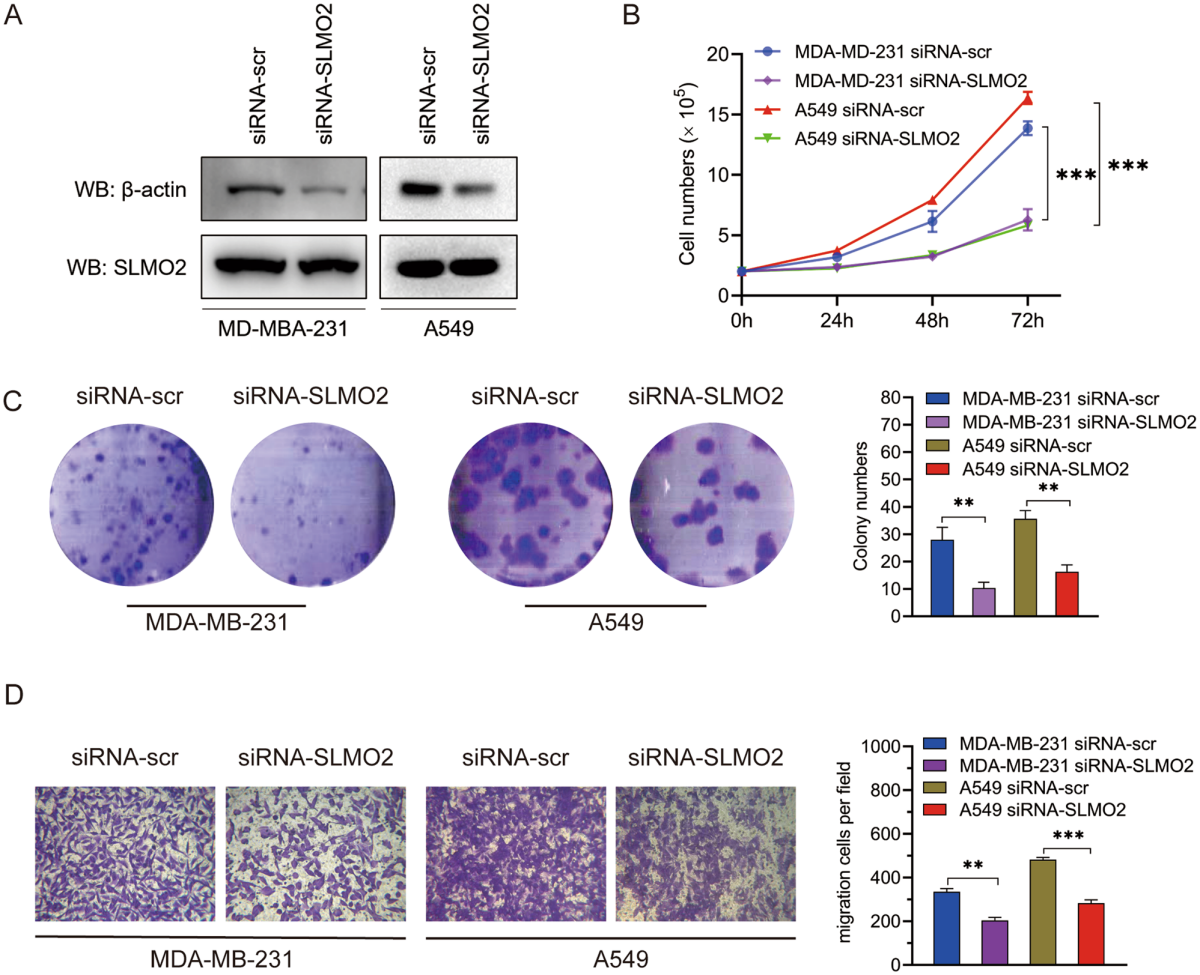
Effects of SLMO2 on proliferation and migration of MDA-MB-231 and A549 cells in vitro. (**A**) Western blot was used to detect siRNA-SLMO2 infection in MDA-MB-231 and A549 cells. (**B**) Proliferation assay was used to evaluate cell proliferation in MDA-MB-231 and A549 cells. (**C**) Clone formation assay was performed to evaluate the growth of MDA-MB-231 and A549 cells. (**D**) Transwell assays were performed to assess cell migration in MDA-MB-231 and A549 cells.

The original Article has been corrected.

