# Supplementary material for: SLMO2 is a potential prognostic and immunological biomarker in human pan-cancer
Source: Sci Rep. 2024 Jan 11;14:1070. doi: 10.1038/s41598-024-51720-5 (PMC10784594; doi:10.1038/s41598-024-51720-5)
Supplement: Supplementary file 1 — Supplementary Information 1. [file 41598_2024_51720_MOESM1_ESM.pptx]

## Slide 1
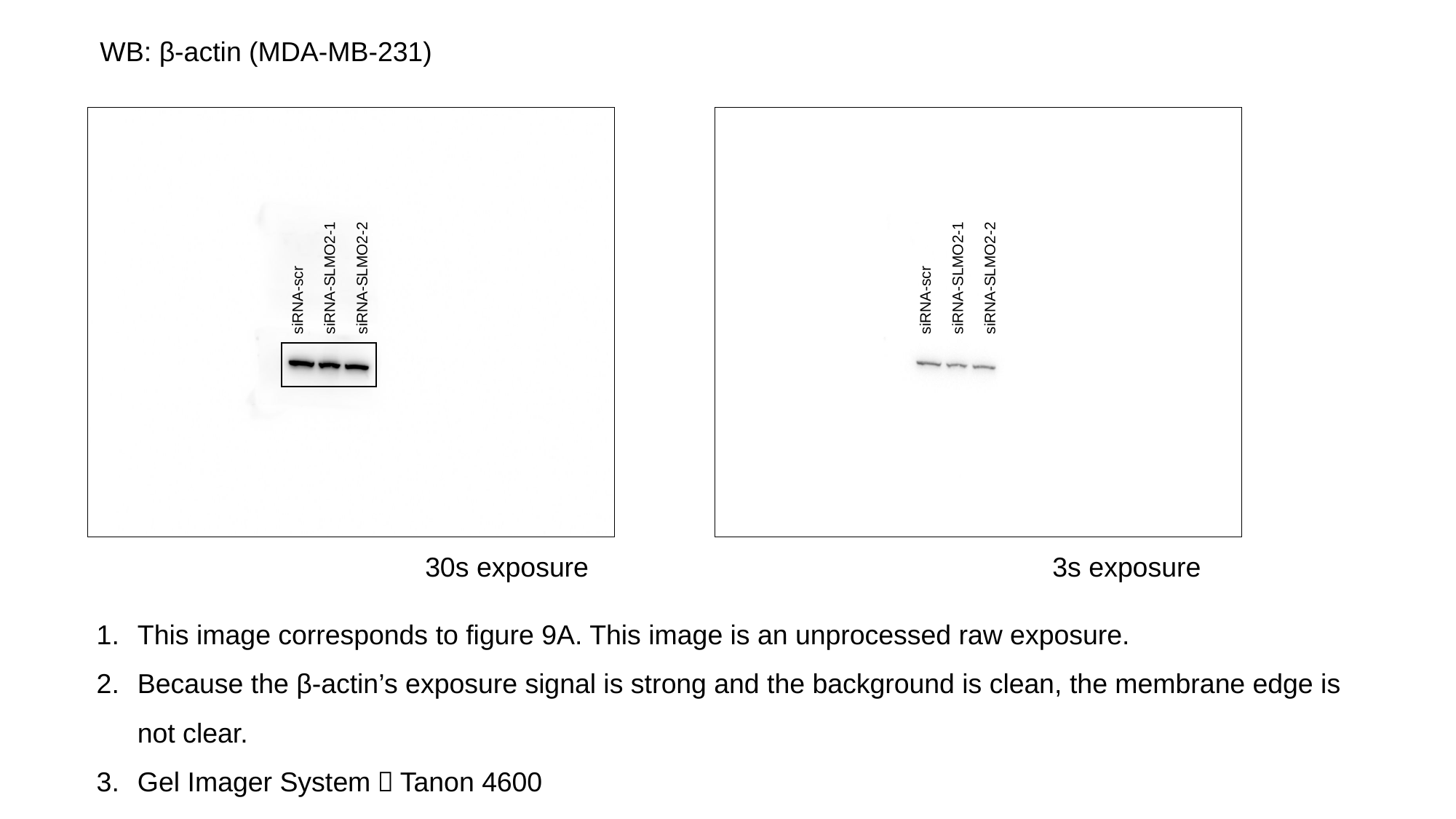

WB: β-actin (MDA-MB-231)
siRNA-SLMO2-1
siRNA-SLMO2-2
siRNA-SLMO2-1
siRNA-SLMO2-2
siRNA-scr
siRNA-scr
30s exposure
3s exposure
This image corresponds to figure 9A. This image is an unprocessed raw exposure.
Because the β-actin’s exposure signal is strong and the background is clean, the membrane edge is not clear.
Gel Imager System：Tanon 4600

## Slide 2
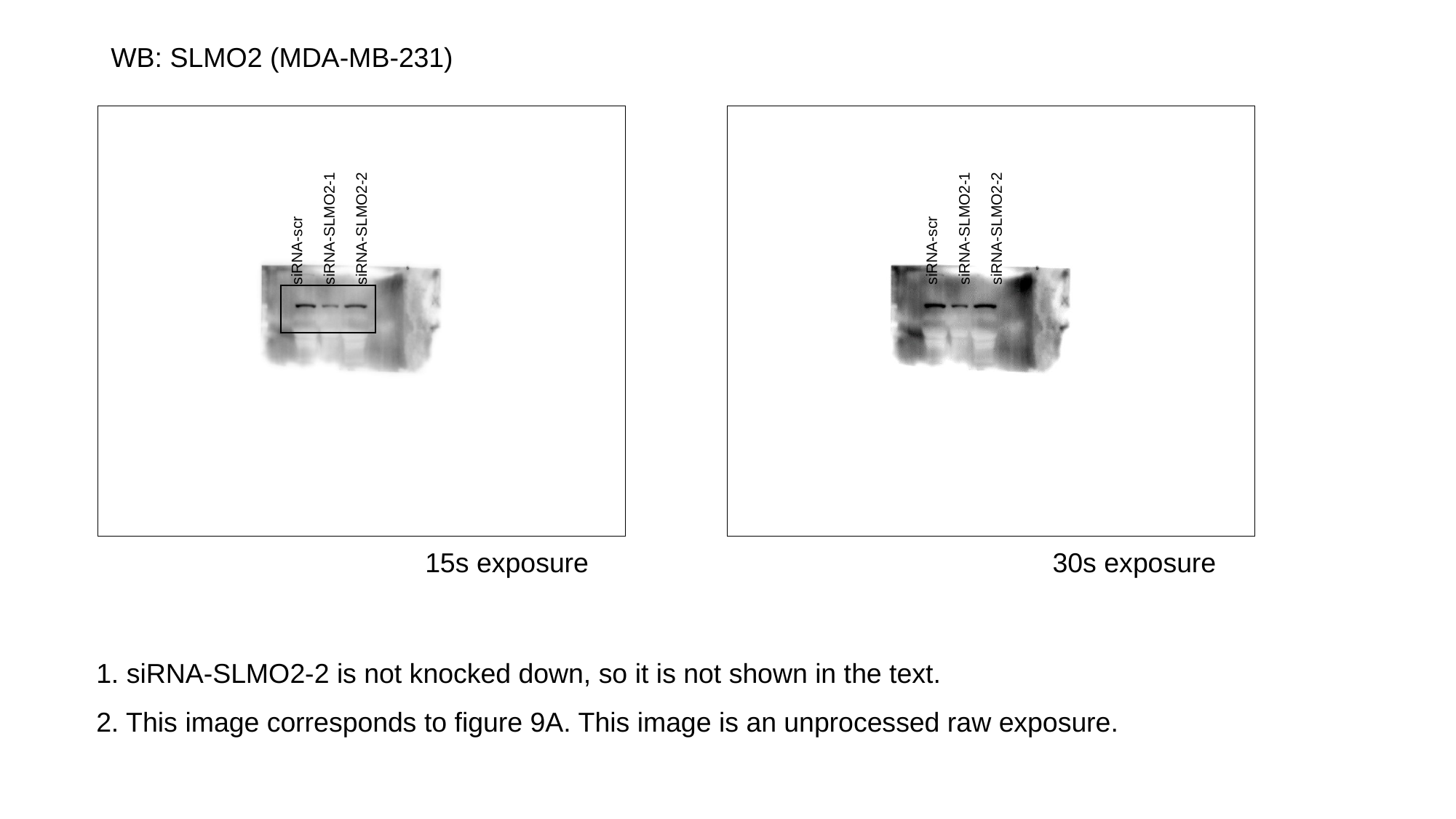

WB: SLMO2 (MDA-MB-231)
siRNA-SLMO2-1
siRNA-SLMO2-2
siRNA-SLMO2-1
siRNA-SLMO2-2
siRNA-scr
siRNA-scr
15s exposure
30s exposure
1. siRNA-SLMO2-2 is not knocked down, so it is not shown in the text.
2. This image corresponds to figure 9A. This image is an unprocessed raw exposure.

## Slide 3
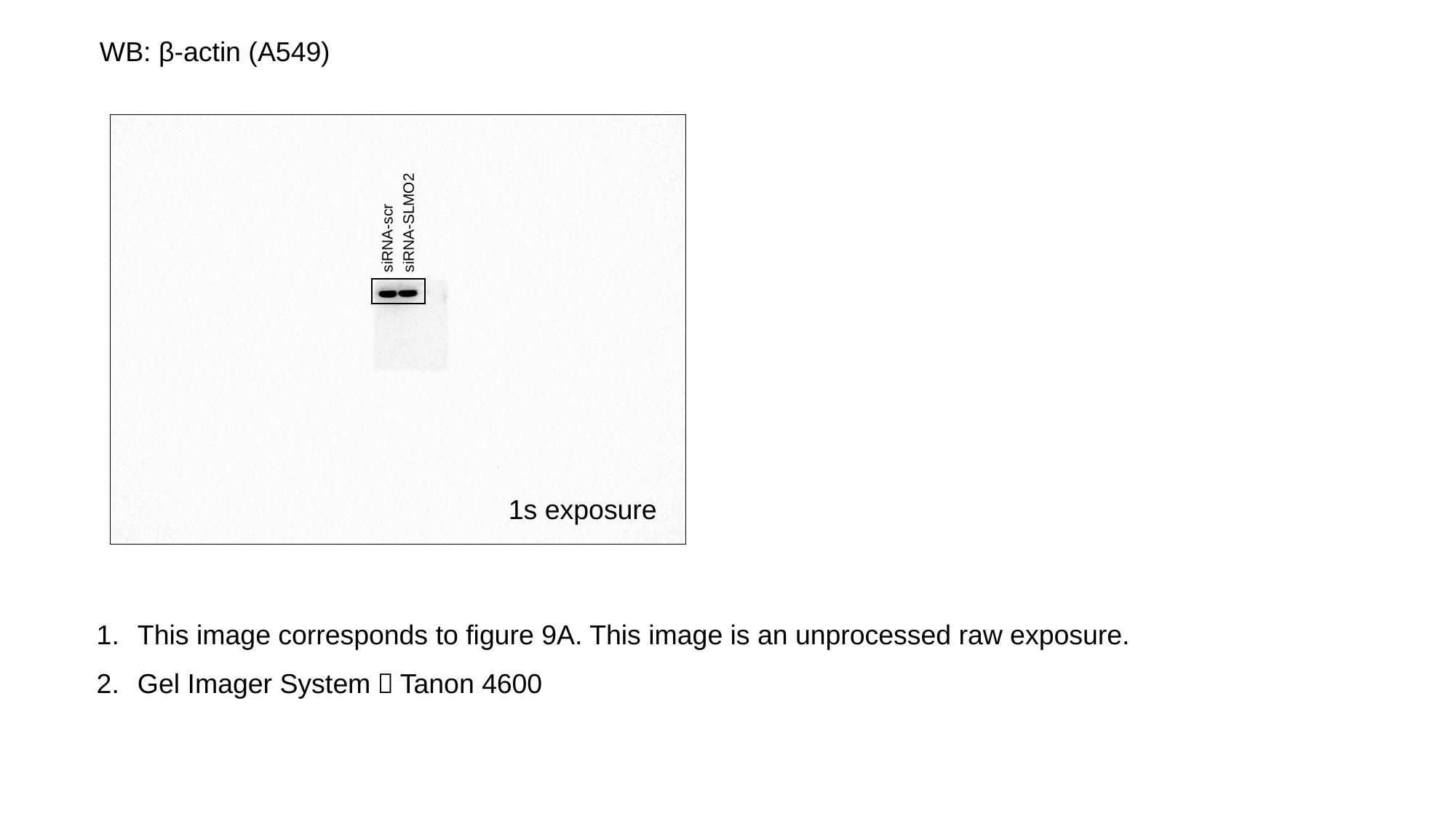

WB: β-actin (A549)
siRNA-SLMO2
siRNA-scr
1s exposure
This image corresponds to figure 9A. This image is an unprocessed raw exposure.
Gel Imager System：Tanon 4600

## Slide 4
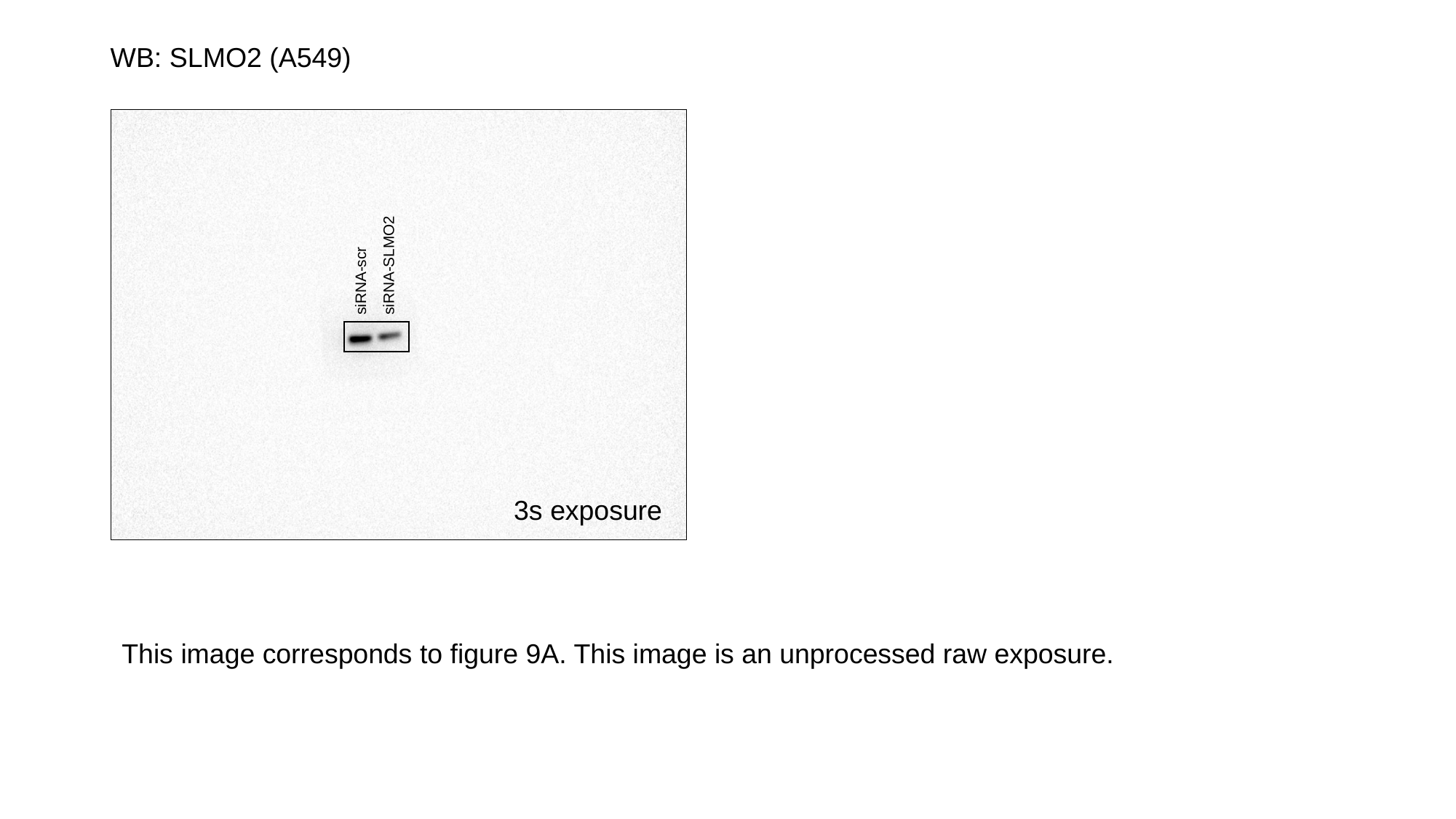

WB: SLMO2 (A549)
siRNA-SLMO2
siRNA-scr
3s exposure
This image corresponds to figure 9A. This image is an unprocessed raw exposure.

## Slide 5
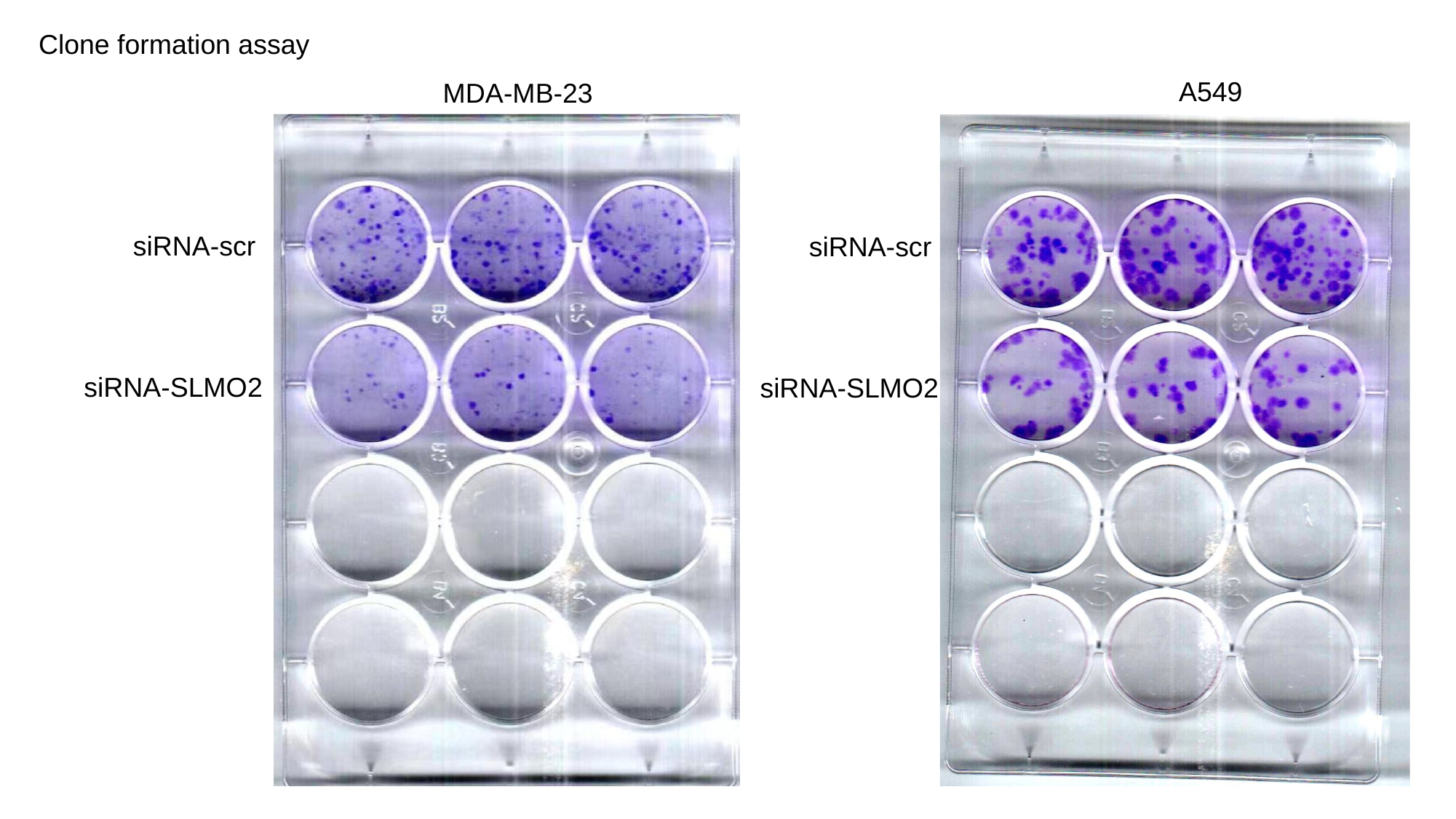

Clone formation assay
A549
MDA-MB-23
siRNA-scr
siRNA-scr
siRNA-SLMO2
siRNA-SLMO2

## Slide 6
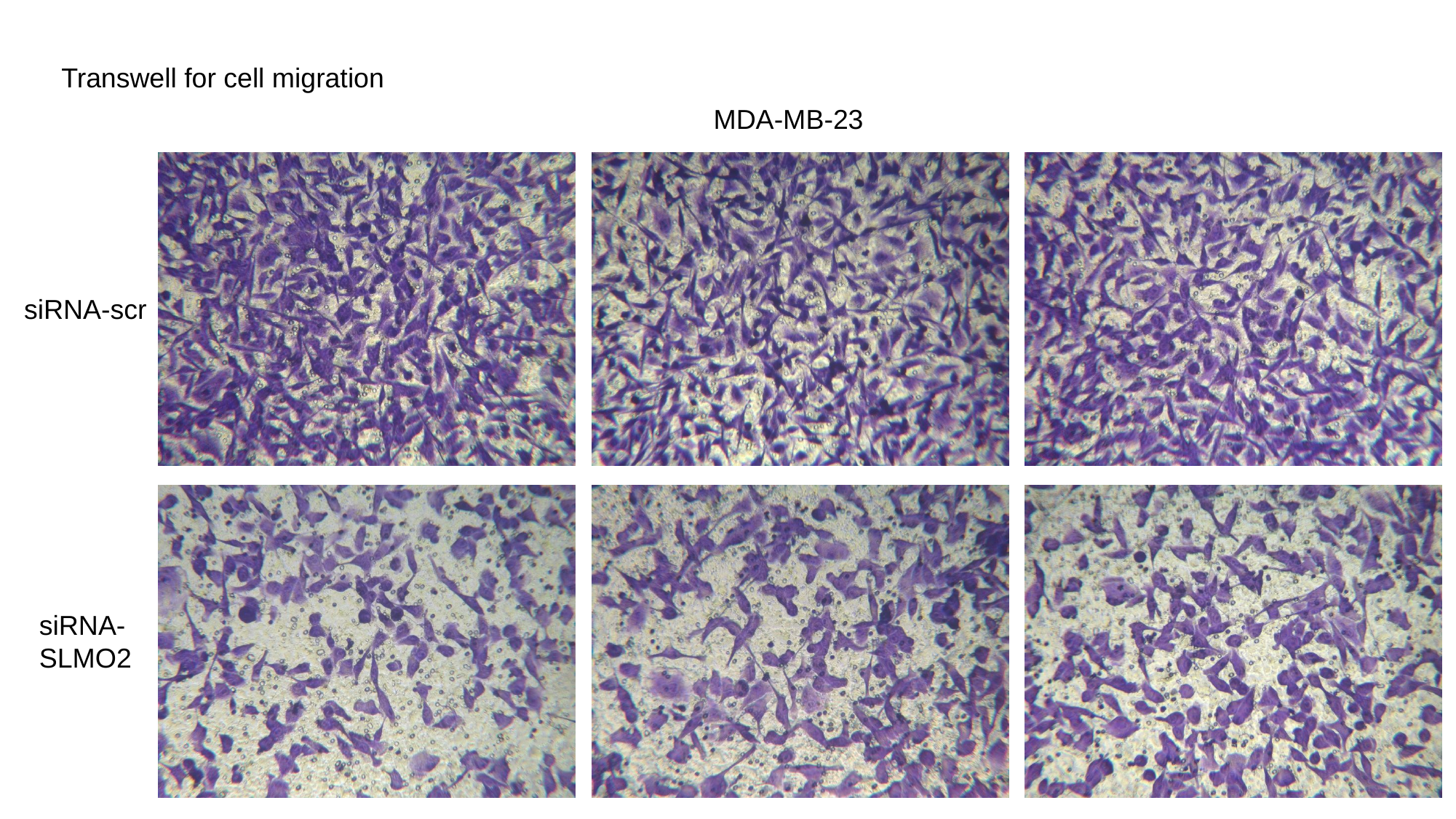

Transwell for cell migration
MDA-MB-23
siRNA-scr
siRNA-
SLMO2

## Slide 7
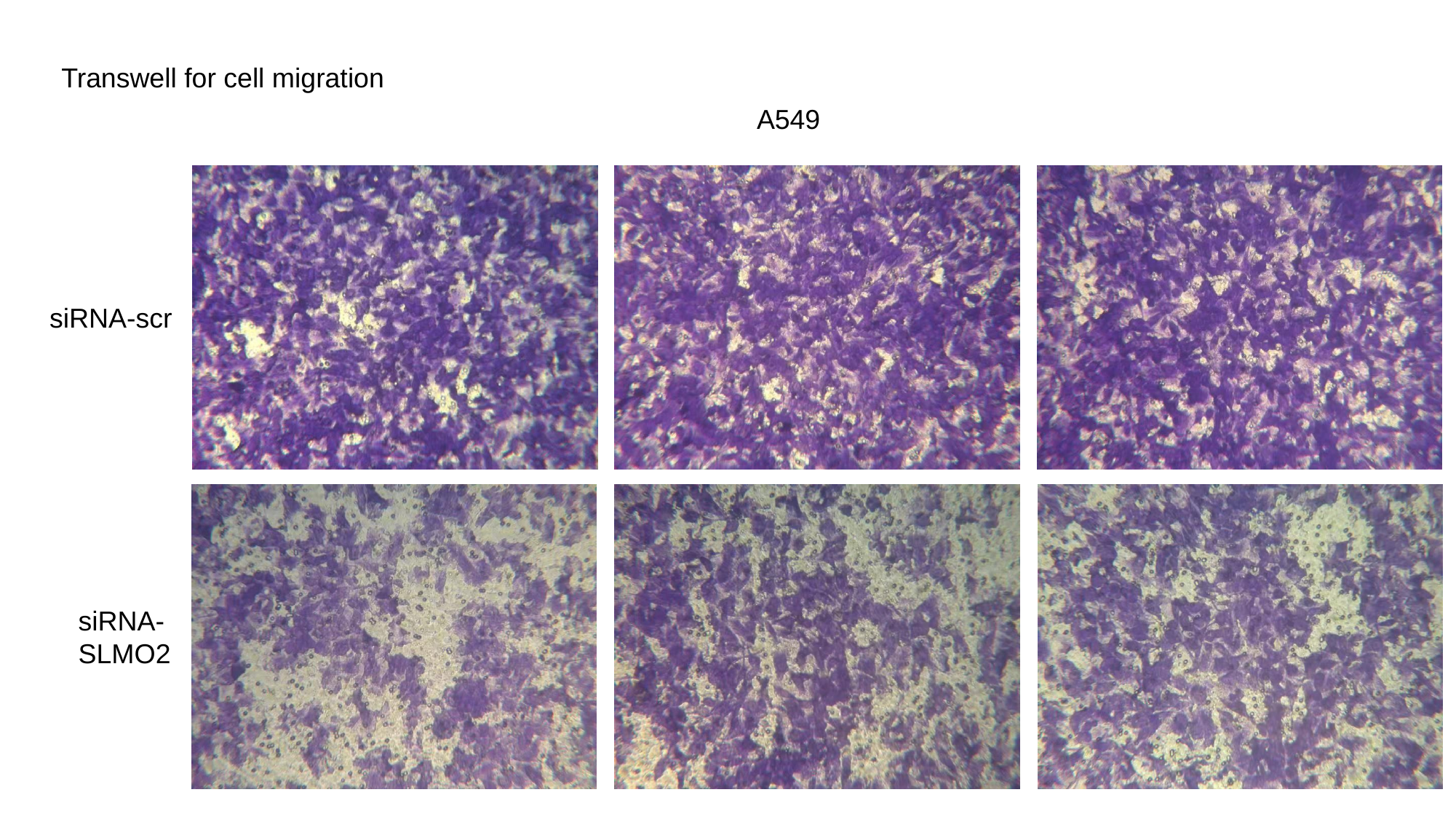

Transwell for cell migration
A549
siRNA-scr
siRNA-
SLMO2
