# Supplementary material for: SLMO2 is a potential prognostic and immunological biomarker in human pan-cancer
Source: Sci Rep. 2024 Jan 11;14:1070. doi: 10.1038/s41598-024-51720-5 (PMC10784594; doi:10.1038/s41598-024-51720-5)
Supplement: Supplementary file 2 — Supplementary Information 2. [file 41598_2024_51720_MOESM2_ESM.docx]

**SLMO2 Is a Prognostic and Immunological Biomarker in Pan-Cancer**

# Supplementary data

**mRNA Expression of *SLMO2*** **in Human Pan-Cancer**

In order to verify the accuracy of the results, we further analyzed the data of the Cancer Cell Line Encyclopedia (CCLE) database. Consistent with previous data, the CCLE data further confirmed that SLMO2 was highly expressed in most tumors (Fig. S1A and 1B).

#
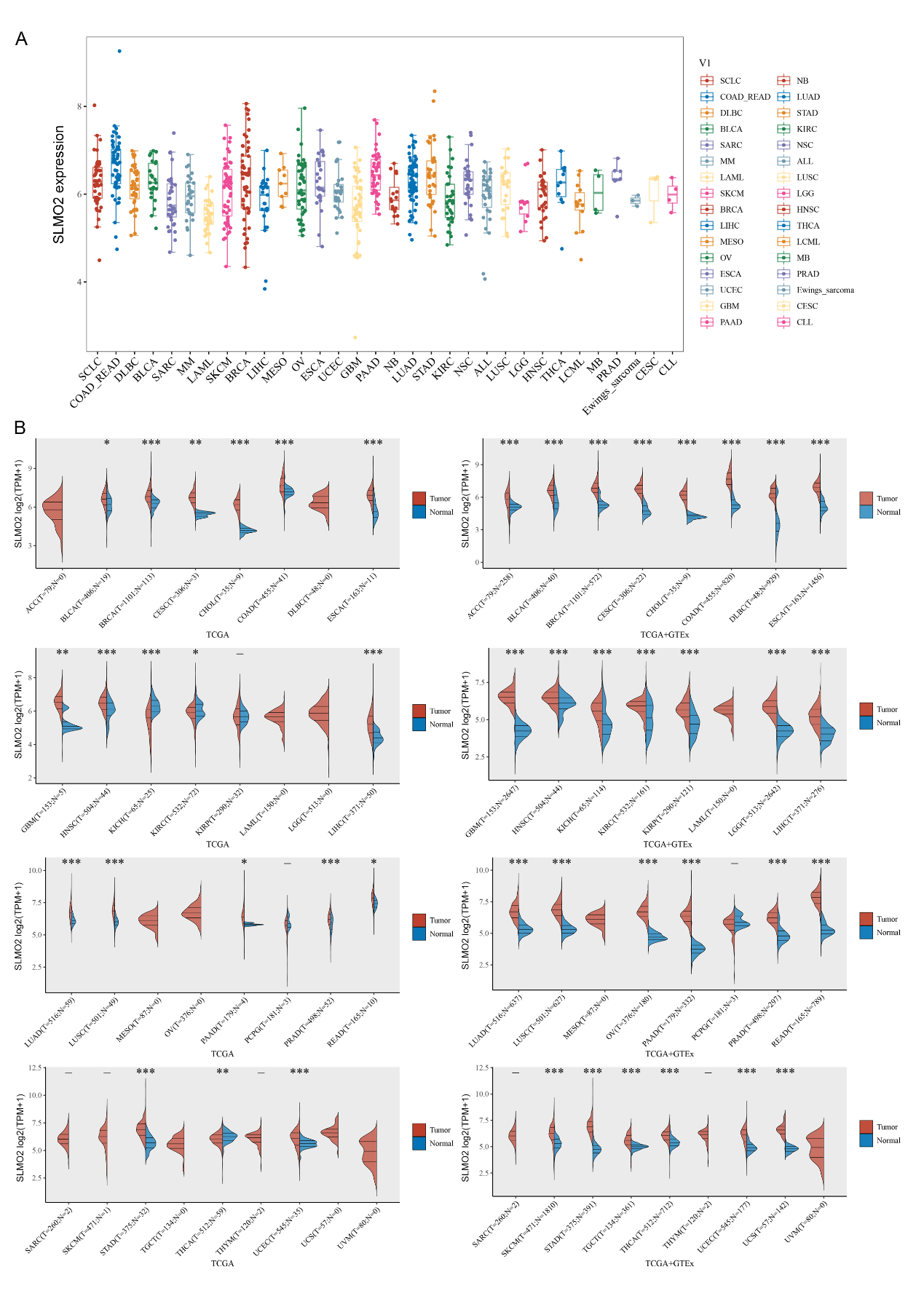


**Figure S1: mRNA Expression of SLMO2 in Human Pan-Cancer.** (A) mRNA expression levels of SLMO2 were analyzed in different cancer types from TCGA data in CCLE. (B) Differences of SLMO2 expression between cancers from the TCGA database and normal samples from the GTEx database. * P < 0.055, ** P < 0.01, *** P < 0.001.

**Relationship between DNA Methylation Levels of SLMO2 and Prognosis.**

We analyzed nine methylation probes associated with SLMO2 in the MethSurv database, including: cg02912129, cg03255221, cg06943251, cg08363339, cg12102151, cg14073986, cg20623172, cg20726575, and cg26216876 (Table S1). Meanwhile, we analyzed the correlation between SLMO2 DNA methylation and prognosis in different tumors (Fig. S2). The results suggest that SLMO2 patients with high DNA methylation level have better prognosis. SLMO2 may affect the prognosis of cancer patients through methylation.

| Cancer | Probe Name | HR | p-value |
| --- | --- | --- | --- |
| ACC | cg06943251 | 0.441 | 0.041426307 |
| BLCA | cg26216876 | 1.375 | 0.033247562 |
| CESC | cg12102151 | 1.679 | 0.033502348 |
| COAD | cg14073986 | 1.996 | 0.043574818 |
| ESCA | cg20726575 | 0.591 | 0.026541841 |
|  | cg20623172 | 0.611 | 0.036200497 |
| GBM | cg03255221 | 0.555 | 0.01066171 |
|  | cg26216876 | 0.54 | 0.011440969 |
|  | cg08363339 | 0.53 | 0.014159213 |
|  | cg14073986 | 0.559 | 0.022306774 |
| HNSC | cg06943251 | 1.398 | 0.013618696 |
|  | cg20623172 | 1.379 | 0.028059067 |
| KIRC | cg12102151 | 0.353 | 0.000236814 |
|  | cg06943251 | 0.363 | 0.000499157 |
|  | cg02912129 | 1.912 | 0.02013657 |
|  | cg08363339 | 0.548 | 0.023648475 |
|  | cg20623172 | 1.549 | 0.027254988 |
| KIRP | cg20623172 | 0.23 | 8.77E-06 |
| LAML | cg26216876 | 0.542 | 0.010187366 |
| LGG | cg26216876 | 0.324 | 8.68E-10 |
|  | cg08363339 | 0.375 | 7.58E-08 |
|  | cg14073986 | 0.523 | 0.000557209 |
|  | cg20623172 | 0.422 | 0.000967047 |
|  | cg03255221 | 0.652 | 0.02358491 |
| LIHC | cg20623172 | 0.661 | 0.018326912 |
| LUAD | cg20726575 | 0.621 | 0.006110251 |
| LUSC | cg12102151 | 0.589 | 0.001363528 |
| PAAD | cg20726575 | 0.55 | 0.009519293 |
| SARC | cg08363339 | 0.567 | 0.008734366 |
|  | cg02912129 | 1.544 | 0.03330106 |
| STAD | cg14073986 | 1.469 | 0.030208253 |
|  | cg20623172 | 1.384 | 0.049755907 |
| UCEC | cg12102151 | 1.946 | 0.033388968 |
|  | cg08363339 | 0.586 | 0.036334498 |
| UVM | cg12102151 | 0.342 | 0.011326871 |

**Table S1: Relationship between DNA Methylation Levels of SLMO2 and Prognosis.**


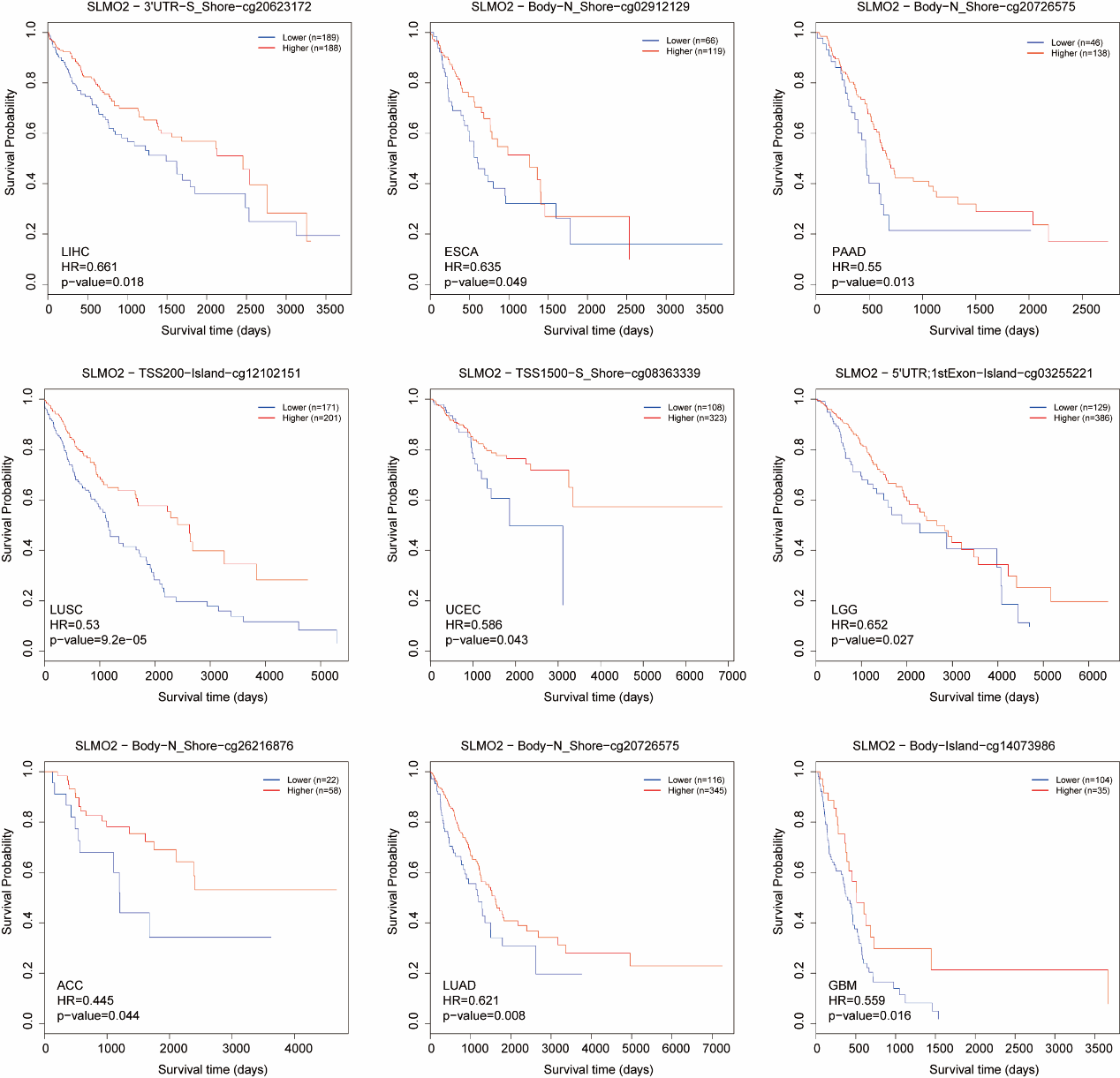


**Figure S2: Relationship between DNA Methylation Levels of SLMO2 and Prognosis.** We used MethSurv to analyze the relationship between DNA methylation level and prognosis of SLMO2

**Correlation Analysis between SLMO2 Expression and Immune Infiltration.**

We analyzed levels of multiple immune cells infiltration across different cancer types in the TIMER2.0 database. As shown in Figures S3 and S4, we found that no significant correlation between SLMO2 expression and other subgroups of immune cell infiltration except MDSC, including B cells, CD4+ T cells, CD8+ T cells, myeloid cells, macrophages, NK cells, Tfh, gamδ T cells, Tregs, monocytes, neutrophil.


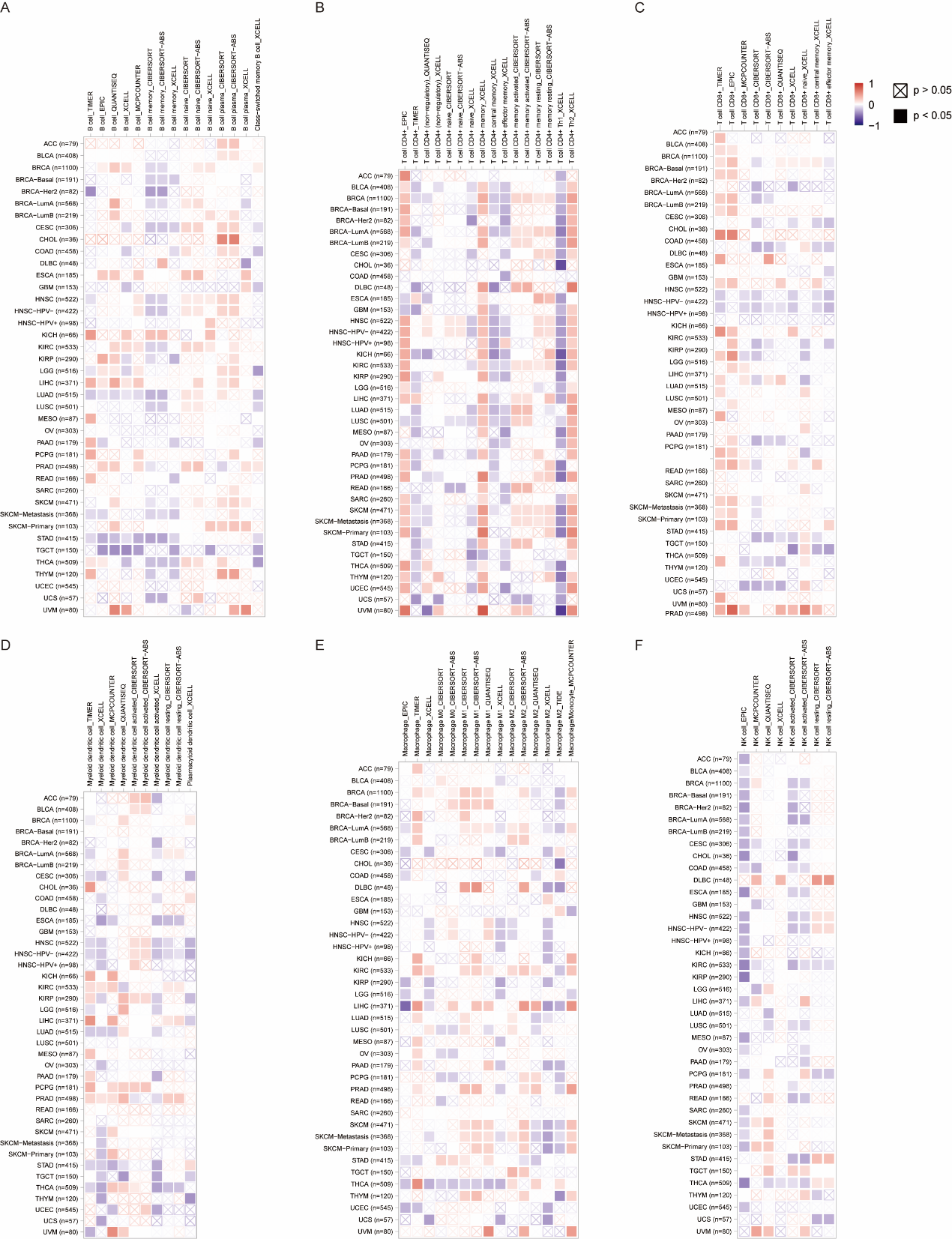


**Figure S3: Correlation analysis between SLMO2 expression and immune infiltration.** We analyzed levels of multiple immune cells infiltration across different cancer types in the TIMER2.0 database, including B cells, CD4+ T cells, CD8+ T cells, myeloid cells, macrophages, NK cells.


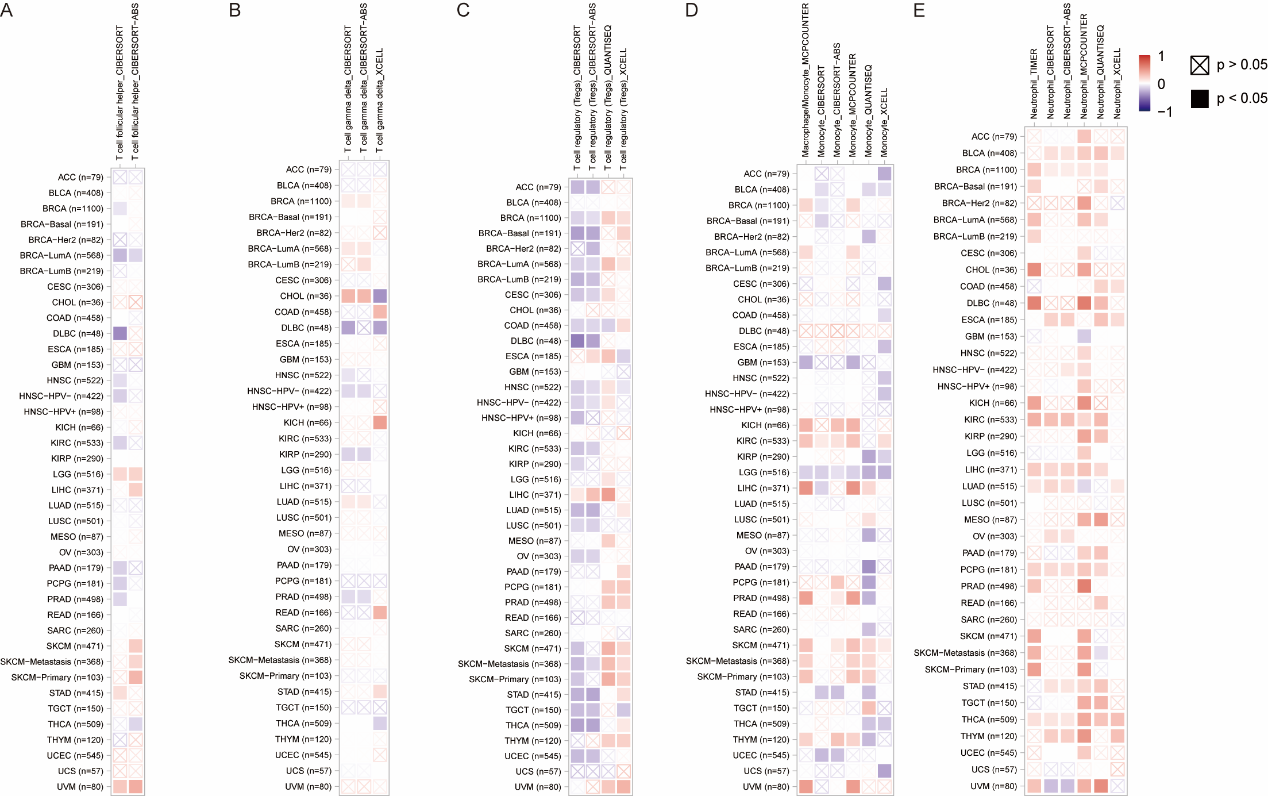


**Figure S4: Correlation analysis between SLMO2 expression and immune infiltration.** We analyzed levels of multiple immune cells infiltration across different cancer types in the TIMER2.0 database, including Tfh, gamma δ T cells, Tregs, monocytes, neutrophil.

**MDSC-Induced Immune Infiltration Predicts a Poor Clinical Outcome in Pan-Cancer.**

We investigated the correlation between MDSC infiltration level and the prognosis of patients with different tumors in the TCGA dataset of TIMER2.0. Our results showed that a high level of MDSC infiltration was associated with a poor prognosis in most tumors (Fig. S5).


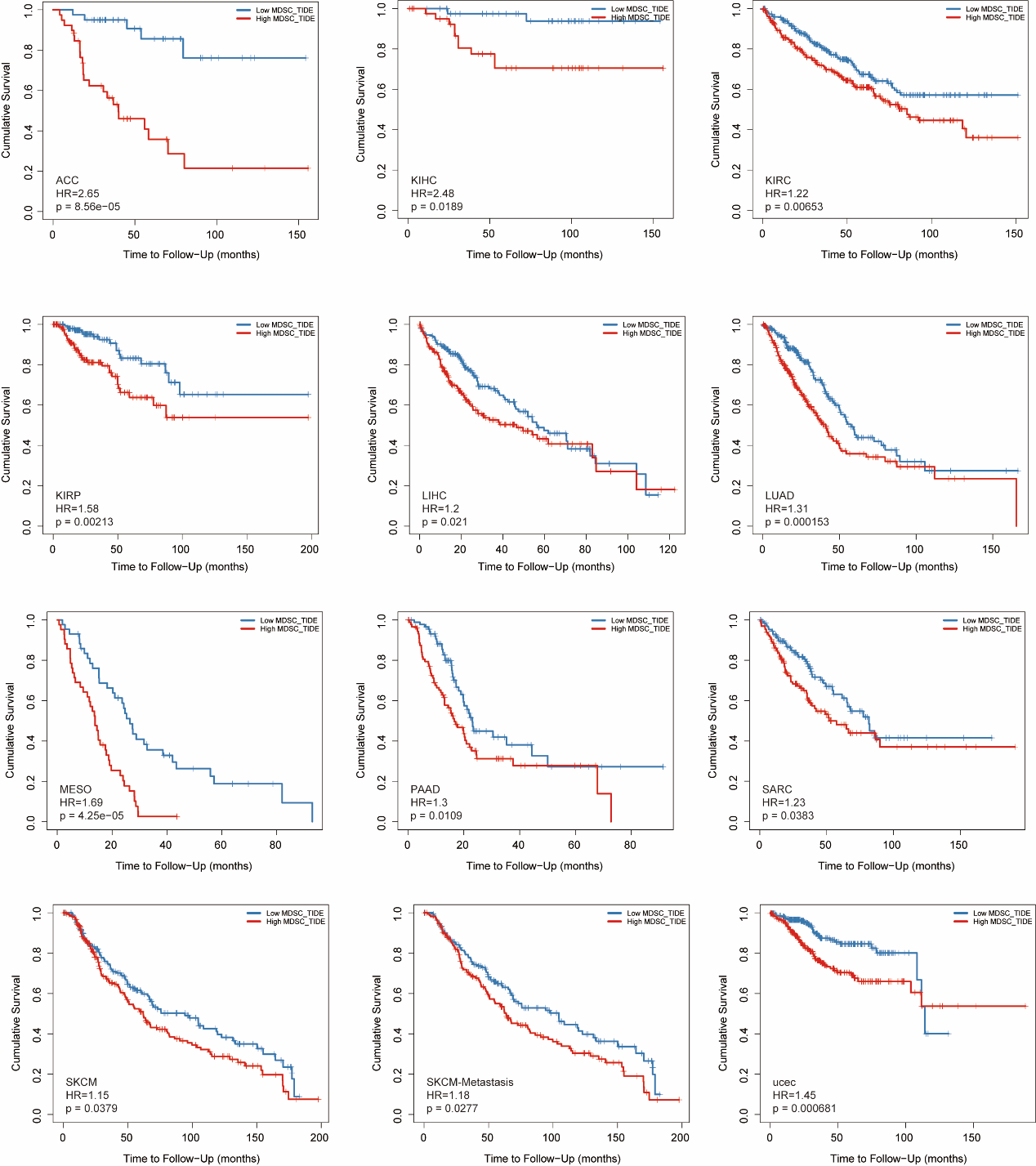


**Figure S5: MDSC-Induced Immune Infiltration Predicts a Poor Clinical Outcome in Pan-Cancer.** Correlation of MDSC infiltration level and the prognosis with different tumors in TCGA data sets in TIMER2.0.

**Table S2. List of primers used for SLMO2 knockdown.**

|  | RNA oligo sequences (5′→3′) |  |
| --- | --- | --- |
| siRNA-1 | AACAAAUAAAUAUAUAGUCAG | sense |
|  | GACUAUAUAUUUAUUUGUUAU | anti-sense |
| siRNA-2 | UAAAACAAAGUACAUUUACUG | sense |
|  | GUAAAUGUACUUUGUUUUAUG | anti-sense |
